# Candidate genes linking maternal nutrient exposure to offspring health via DNA methylation: a review of existing evidence in humans with specific focus on one-carbon metabolism

**DOI:** 10.1093/ije/dyy153

**Published:** 2018-08-17

**Authors:** Philip James, Sara Sajjadi, Ashutosh Singh Tomar, Ayden Saffari, Caroline H D Fall, Andrew M Prentice, Smeeta Shrestha, Prachand Issarapu, Dilip Kumar Yadav, Lovejeet Kaur, Karen Lillycrop, Matt Silver, Giriraj R Chandak, EMPHASIS study group, Lena Acolatse, Meraj Ahmed, Modupeh Betts, Giriraj R Chandak, Harsha Chopra, Cyrus Cooper, Momodou K Darboe, Chiara Di Gravio, Caroline H D Fall, Meera Gandhi, Gail R Goldberg, Prachand Issarapu, Philip James, Ramatoulie Janha, Landing M A Jarjou, Lovejeet Kaur, Sarah H Kehoe, Kalyanaraman Kumaran, Karen A Lillycrop, Mohammed Ngum, Suraj S Nongmaithem, Stephen Owens, Ramesh D Potdar, Andrew M Prentice, Ann Prentice, Tallapragada Divya Sri Priyanka, Ayden Saffari, Sirazul Ameen Sahariah, Sara Sajjadi, Harshad Sane, Smeeta Shrestha, Matt J Silver, Ashutosh Singh Tomar, Kate A Ward, Dilip Kumar Yadav, Chittaranjan S Yajnik

**Affiliations:** 1 MRC Unit The Gambia at the London School of Hygiene and Tropical Medicine, London, UK; 2 Genomic Research on Complex Diseases (GRC Group), CSIR-Centre for Cellular and Molecular Biology, Hyderabad, India; 3 MRC Life course Epidemiology Unit, University of Southampton, Southampton General Hospital, Southampton, UK; 4 School of Basic and Applied Sciences, Dayananda Sagar University, Bangalore, India; 5 Research Centre for Biological Sciences, Institute of Developmental Sciences, University of Southampton, Southampton, UK

**Keywords:** Epigenetics, DNA methylation, fetal programming, Developmental Origins of Health and Disease, one-carbon metabolism, candidate genes, metastable epialleles, cognitive development, cardiometabolic outcomes, growth

## Abstract

**Background:**

Mounting evidence suggests that nutritional exposures during pregnancy influence the fetal epigenome, and that these epigenetic changes can persist postnatally, with implications for disease risk across the life course.

**Methods:**

We review human intergenerational studies using a three-part search strategy. Search 1 investigates associations between preconceptional or pregnancy nutritional exposures, focusing on one-carbon metabolism, and offspring DNA methylation. Search 2 considers associations between offspring DNA methylation at genes found in the first search and growth-related, cardiometabolic and cognitive outcomes. Search 3 isolates those studies explicitly linking maternal nutritional exposure to offspring phenotype via DNA methylation. Finally, we compile all candidate genes and regions of interest identified in the searches and describe their genomic locations, annotations and coverage on the Illumina Infinium Methylation beadchip arrays.

**Results:**

We summarize findings from the 34 studies found in the first search, the 31 studies found in the second search and the eight studies found in the third search. We provide details of all regions of interest within 45 genes captured by this review.

**Conclusions:**

Many studies have investigated imprinted genes as priority loci, but with the adoption of microarray-based platforms other candidate genes and gene classes are now emerging. Despite a wealth of information, the current literature is characterized by heterogeneous exposures and outcomes, and mostly comprise observational associations that are frequently underpowered. The synthesis of current knowledge provided by this review identifies research needs on the pathway to developing possible early life interventions to optimize lifelong health.

**Key Messages**

- The body of evidence linking maternal nutritional exposure to offspring phenotype via DNA methylation in humans is rapidly growing yet currently remains complex and inconsistent.
- Candidate genes in the field of intergenerational nutritional epigenetics go beyond imprinted genes to include other gene classes such as metastable epialleles.
- Going forwards, there is a continued need for adequately powered prospective cohort studies with repeated longitudinal measurements and randomized nutritional interventions to track the full continuum from maternal exposure to offspring epigenotype to later phenotype.

## Introduction

Epigenetic modifications influence gene expression without altering the nucleotide sequence, through the action of a diverse array of molecular mechanisms including DNA methylation, histone modifications and RNA-mediated effects.^1^ Epigenetic processes have been implicated in the aetiology of a variety of diseases,^2^ most prominently cancer^3^ and fetal growth disorders.^4^ Epigenetic marks are mitotically heritable and can be influenced by the environment,^5^ suggesting a potential mechanism linking early life exposures to later phenotype,^6^^,^^7^ a notion supported by animal studies.^8–10^ However, the extent to which epigenetics plays a role in fetal programming in humans remains relatively unexplored. In this review we collate evidence from human intergenerational studies, exploring which nutritional exposures during pregnancy may affect DNA methylation in the offspring, and the possible impact of such modifications on health and disease risk across the life course.

### DNA methylation and gene expression

Many biological processes rely on DNA methylation, including genomic imprinting, X-chromosome inactivation and tissue-specific gene expression.^11^ DNA methylation describes the addition of a methyl group to a cytosine base at the 5’ carbon position to form 5-methylcytosine, catalyzed by DNA methyltransferases (DNMTs). This most commonly occurs at cytosine bases adjacent to guanine, termed CpG (‘cytosine-phosphate-guanine’) sites. Regions of high CpG density are known as ‘CpG islands’, and approximately two-thirds of human genes contain these in their promoter regions.^12^ DNA methylation has been shown to influence transcriptional activity either by blocking transcription factors binding to the DNA, or by the recruitment of histone modifiers which promote a closed chromatin structure and gene silencing.^1^ CpG methylation within promoters is typically associated with transcriptional silencing,^13^ although not consistently, and the effect of DNA methylation may vary depending on which region within the gene is methylated.^14^ There is also increasing evidence that DNA methylation and histone modifications work in concert with non-coding RNAs to regulate gene expression.^15^ DNA methylation plays a role in chromatin remodelling, as DNMT enzymes at CpG sites can be physically linked to enzymes which bring about histone methylation and de-acetylation.^13^ MicroRNAs (miRNAs) affect gene expression through binding to messenger RNAs (mRNAs) and repressing translation,^16^ including mRNAs that control the expression of DNMTs and histone deacetylases.^15^ The transcription of some miRNA classes can be influenced by CpG methylation and histone modifications.^16^

### Epigenetics, windows of plasticity and the Developmental Origins of Health and Disease

The Developmental Origins of Health and Disease (DOHaD) hypothesis posits that early life exposure to environmental insults can increase the risk of later adverse health outcomes.^7^ David Barker’s early cohort studies showed that lower birthweight was associated with an increased risk of hypertension, type 2 diabetes (T2D) and cardiovascular disease in later life,^17^ findings that were widely replicated.^18^ Risk of disease was further exacerbated by rapid childhood weight gain, adult obesity and other lifestyle factors such as unhealthy diets, smoking and lack of exercise.^19^^,^^20^ The Dutch Hunger Winter studies showed that exposure to famine during pregnancy was associated with a wide range of phenotypes in the adult offspring, including increased blood pressure,^21^ obesity^22^ and schizophrenia,^23^ effects that depended on the timing of the exposure during pregnancy.^22^

Epigenetic processes are emerging as potential mechanisms to explain these and other associations found in the DOHaD literature. For example the ‘thrifty epigenome’ hypothesis proposes that *in utero* exposures can shape an epigenetic signature, resulting in a phenotype that is ‘adapted’ to the early life environment but which may prove to be ‘maladapted’ if the environment changes in later life.^24^ Therefore famine exposure during pregnancy could programme ‘thrifty epigenotypes’ that are adapted to a nutritionally poor environment, but this may subsequently trigger metabolic disease if the adult environment changes to one that is nutritionally abundant.

The periconceptional period is a time of rapid cell differentiation and epigenetic remodelling, and may therefore represent a critical window during which the developing epigenome is sensitive to environmental influences.^25^ We define the periconceptional window from 14 weeks preceding conception until 10 weeks after conception.^26^ Within 48 hours of fertilization, there is rapid erasure of methylation marks to render the developing cells pluripotent.^11^ After implantation, re-methylation occurs in a tissue-specific manner, and continues throughout pregnancy, enabling differentiation of somatic cells. A second wave of demethylation occurs in the primordial germ cells as they migrate to the genital ridge.^27^ At this stage most parental imprints are erased, so that sex-specific imprints can be laid down. In boys the prospermatogonia then undergo re-methylation throughout gestation, whereas in girls the oocytes continue to be re-methylated over the duration of their maturation, with evidence of high activity as each egg ripens before ovulation.^27^

Notable classes of loci that may be especially sensitive to early environmental exposure include imprinted genes, metastable epialleles (MEs) and transposable elements (TEs).^6^ Imprinted genes exhibit monoallelic expression, whereby only the maternally or paternally inherited allele is expressed, with expression controlled by regulatory regions whose methylation state is inherited in a parent of origin-specific manner.^28^ MEs are genomic loci showing variable methylation between individuals, but showing high correlation in methylation status across tissues within the same individual, indicating establishment of methylation state in the first few days after conception, preceding gastrulation.^29^ MEs therefore help to pinpoint the timing of an exposure influencing ME methylation to the periconceptional period.^30^^,^^31^ TEs are small, mobile sequences of DNA that are thought to comprise 45% of the human genome.^32^ They can insert into new genomic locations and become disruptive if transposed into a functional gene or when increasing copy number. Whereas most TEs are silenced epigenetically,^33^ some have variable methylation patterns that have been shown to be influenced by nutrition in mice.^9^ Their methylation states can alter neighbouring gene expression, exemplified by the Agouti mouse model detailed later.

### Influence of nutrition on DNA methylation

**Figure 1.**
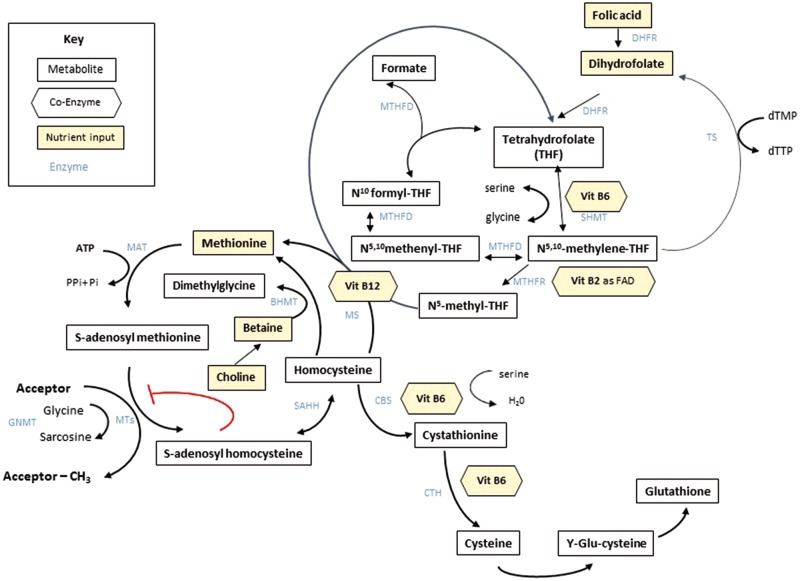
A simplified summary of one-carbon metabolism. BHMT, Betaine Homocysteine MethylTransferase; CBS, Cystathionine-Beta-Synthase; CTH, Cystathionine Gamma-Lyase; DHFR, Dihydrofolate Reductase; dTMP, Deoxythymidine Monophosphate; dTTP, Deoxythymidine Triphosphate; FAD, Flavin Adenine Dinucleotide; GNMT, Glycine N-MethylTransferase; MAT, Methionine AdenosylTransferase; MS, Methionine Synthase; MT, Methyl Transferases; MTHFD, MethyleneTetraHydroFolate Dehydrogenase; MTHF, MethyleneTetraHydroFolate Reductase; SAHH, S-Adenosyl Homocysteine Hydrolase; SHMT, Serine HydroxyMethylTransferase; TS, Thymidylate Synthase. Source: reproduced with permission from James *et al.* Epigenetics, nutrition and infant health. In: Karakochuk C, Whitfield K, Green T, Kraemer K (eds). *The Biology of the First 1000 Days*. Boca Raton, FL: CRC Press, 2017.

A range of maternal exposures have been associated with DNA methylation including nutrition, stress, infection, pollutants, smoking, radiation, level of exercise and parental body composition.^34–36^ Animal studies suggest that the epigenome is particularly sensitive to such environmental factors in early life, notably during the prenatal and neonatal periods.^9^^,^^25^^,^^37^ Studies of the effects of early life nutrition on DNA methylation have shown that maternal under- or over-nutrition or differences in protein, fat, sugar or micronutrient intake during gestation can induce epigenetic and phenotypic changes in the offspring.^8^^,^^38^ Recent studies have also shown that variations in paternal diet or body composition might also induce long-term epigenetic and phenotypic changes in the offspring.^39^ One-carbon nutrients and metabolites are thought to be particularly important in the periconceptional period and during embryonic development.^26^ One-carbon metabolism (OCM) pathways link the folate, methionine, homocysteine, transsulphuration and transmethylation metabolic pathways together (Figure 1). These are crucial for many biochemical processes, including DNA methylation.

Nutrition plays a key role in OCM by providing substrates (folate, methionine, choline and betaine) and essential co-factors (vitamins B12, B6 and B2). For example, B12 is required by methionine synthase to methylate homocysteine, B6 is essential in the homocysteine transsulphuration pathway, and both B6 and B2 are needed to reduce dietary folate to methyltetrahydrofolate. A more detailed overview of OCM and the role of nutrients in these pathways is provided in Supplementary Material 1, available as Supplementary data at *IJE* online.

The potential for maternal nutrition to both alter offspring DNA methylation and influence phenotype is famously illustrated by the Agouti mouse experiments. Two groups of pregnant dams were fed diets that differed only in nutrients essential to OCM (folic acid, choline, betaine and B12). Increased levels of one-carbon nutrients increased methylation in the isogenic pups at a retrotransposon locus [Intracisternal A Particle (IAP), also an ME] upstream of the Agouti gene. The degree of expression of the Agouti gene depended on the level of IAP methylation, and this in turn altered the pups’ fur colour, as well as their appetite, adiposity and glucose tolerance in adulthood.^6^^,^^9^

## Review methodology

We performed a narrative review of the literature in three stages to form the thematic analysis in this paper. First we searched for studies describing associations between preconceptional or pregnancy nutritional exposures and DNA methylation in offspring. We limited this search to human studies that used an intergenerational design. We included nutritional exposures in dietary or supplemental form related to OCM, or broader measures that could influence availability of such nutrients (famine, seasonal diets and macronutrients). We excluded paternal exposures and nutrients not directly involved in OCM, and we only considered epigenetic studies focusing on DNA methylation. Second, we searched for human studies linking infant DNA methylation to a subset of phenotypic outcomes (growth-related, cardiometabolic and cognitive), restricting the included studies to those describing methylation at genetic loci identified in the first search (‘nutrition-sensitive’ loci). Third, we isolated those studies explicitly linking maternal nutritional exposure to offspring phenotype via DNA methylation. Three authors (P.J., S.S., A.S.T.) performed the searches in PubMed and Google Scholar, assessing titles and abstracts against the inclusion criteria. Reference sections of included studies and relevant review papers were also used to help confirm that key studies had been included. Searches took place from January to March 2017. Supplementary Material 2, available as Supplementary data at *IJE* online, details the strategy and gives an example of the search terms used in PubMed.

## Review of studies linking maternal nutritional exposure to offspring DNA methylation

We provide a broad overview of the associations found in the literature between maternal nutritional exposure and offspring DNA methylation in Table 1. Below we briefly review the associations by type of exposure, but refer the reader to detailed information on the individual studies (*n*  =  34) in Supplementary Table 1, available as Supplementary data at *IJE* online, which includes information on the nutritional exposures, timing of exposures, study design, DNA tissue, age of offspring and associated genes. All gene names are defined in Table 4 (see candidate gene data summary, below).

**Table 1.** Summary of associations between maternal one-carbon metabolites and broader nutritional exposures with offspring DNA methylation

Timing of exposure	Maternal exposure^a^	Offspring DNA methylation association
		(↑/↓: increased/decreased methylation)
Periconception	*↑*B2	*↑PLAGL1* (*ZAC1)*,^40^*↑VTRNA2-1*^41^
	*↑*Betaine	↑ *DNMT1*,^42^*↑POMC*,^43^*↑RXRA*^44^
	Famine	*↓IGF2*,^45^*↑↓*^b^*IGF2*,^46^*↓INSIGF*,^46^^,^^47^*↑IL10*,^47^*↑GNASAS*,^47^*↑LEP*,^47^*↑ABCA^1^*,^47^*↑MEG3*,^47^*↑TACC1*,^48^*↑ZNF385A*,^48^*↓TMEM105*,^48^*↑PAX8*,^49^*↓*ZFP57,^4^*, ↓PRDM9*^49^
	*↑*Folates	*↓STX11*,^50^*↓OTX2*,^50^*↓TFAP2A*,^50^*↓CYS1*,^50^*↓LEP*,^44^*↑RXRA*^44^
	*↑*Folic acid	*↑LEP*,^42^*↓H19*,^51^*↑IGF2*,^52^*↓IGF2*^44^
	*↑*Multiple micronutrients	*↓GNASAS*,^53^*↓MEG3*,^53^*↓IGF2R*,^53^*↓MEST*^53^
	Seasonality of one-carbon metabolites^c^	*↑POMC*,^43^*↑VTRNA2-*1,^41^*↑BOLA3*,^30^*↑FLJ20433*,^30^*↑PAX8*,^30^*↑SLITRK1*,^30^*↑ZFYVE28*,^30^*↑RBM46*^31^

1st and 2nd trimester	*↑B6*	*↑MEG3*^54^
	*↑*Betaine	*↓ LEP*^42^
	*↑*Carbohydrates	*↓RXRA*^55^
	*↑*Choline	*↓DNMT1*^42^
	Famine	*↑FAM150B*,^48^*↑SLC38A2*,^48^*↑PPAP2C*,^48^*↓OSBPL5/MRGPRG*,^48^*↑TACC1*,^48^* ↑ZNF385A*,^48^*↑PAX8*,^49^*↓ZFP57*,^49^*↓PRDM9*^49^
	*↑*Folates	*↓PEG3*,^56^*↑NR3C1*,^57^*↓MEG3*,^56^*↓PLAGL1*,^56^*↑IGF2*,^56^*↓LEP*,^42^*↓DNMT1*^42^
	*↑*Folic acid	*↓PEG3*,^58^*↑IGF2*,^58^*↓DNMT1*^44^

3rd trimester	*↑B2*	*↑PLAGL1* (*ZAC1)*^40^
	*↑B12*	*↓IGF2*^59^
	*↑*Choline	*↑↓*^d^*NR3C1*,^60^*↑↓*^d^*CRH*,^60^*↑DNMT1*,^42^^,^^44^
	Famine	*↓GNASAS*,^47^*↑TACC1*,^48^*↑ZNF385A*,^48^*↑PAX8*,^49^*↓ZFP57*,^49^*↓PRDM9*^49^
	*↑*Folates	*↑DNMT1*,^44^*↓RXRA*,^42^*↑LASP1*,^61^*↑ACADM*,^61^*↑WNT9A*,^61^*↑FZD7*,^61^*↓ZFP57*,^61^*↓*LY6E,^61^*↓C21orf56*^61^
	*↑*Folic acid	*↑RXRA*^42^
	*↑* Meat and fish intake	*↑HSD2*^62^
	*↑* High sugar, high fat diet	*↑IGF2*^63^
	*↑*Omega-3 PUFA	*↓H19*,^64^*↑IGF2*,^6^ mostly *↓*associations in EWAS^65^
	*↑*Omega-6 PUFA	*↓MIRLET7BHG*^66^

a Like nutrients are shaded in the same colour during each time period.

b Different associations at different loci within gene.

c Rainy season (higher concentration of most one-carbon metabolites) versus dry season.

d Different associations between different tissues.

EWAS, epigenome-wide association study; PUFA, polyunsaturated fatty acids.

### Folate

Associations between maternal folate exposure and the offspring methylome are inconsistent, with varying effects according to the form of folate (dietary folates or folic acid supplements)^58^ the timing of exposure,^42^^,^^58^ baseline maternal folate status,^50^^,^^61^ underlying genotype,^67^ the genomic region affected^68^ and individual CpG site.^42^

Periconceptional folic acid has been positively associated with offspring methylation at *LEP*,^42^ inversely associated with methylation at *H19*,^51^ and has demonstrated both positive^52^ and inverse^44^ associations at *IGF2*. Not all studies have found an effect of periconceptional folic acid exposure.^58^ Supplementation started after 12 weeks of gestation has been associated with increased offspring methylation at *IGF2* and decreased methylation at *PEG3.*^58^ Folic acid taken up to the end of the second trimester has been inversely associated with *DNMT1* methylation, but positively correlated at the same locus when the folic acid consumption was extended into the third trimester.^44^

Data for dietary folate intakes (assessed using questionnaires or plasma samples) are equally variable. Periconceptional folate intake and offspring DNA methylation were inversely associated with the majority of differentially methylated CpGs in an epigenome-wide screen, although this trend reversed in stratified analysis among women with low intakes (<200  µg/day).^50^ Periconceptional intakes have also been inversely associated with methylation at *LEP* and positively associated at *RXRA.*^44^ First trimester folate exposure has shown positive associations with DNA methylation at *IGF2*^56^ and *NR3C1*,^57^ and inverse associations at *MEG3*, *PLAGL1* and *PEG3.*^56^ For second trimester folate exposure, studies have reported inverse associations at multiple differentially methylated CpG sites,^68^ and at *LEP* and *DNMT1.*^42^ Third trimester folate exposure has shown positive associations with methylation at *DNMT1*,^44^ and at *LASP1*, *ACADM*, *WNT9A*, *C21orf56* and *FZD7*,^61^ but inverse associations at *ZFP57*, *LY6E* and *RXRA.*^42^^,^^61^

### B vitamins

Maternal serum B12 at first antenatal visit has been inversely associated with cord blood global methylation levels,^67^ and inversely associated with offspring *IGF2* methylation when exposure timing was at delivery.^59^ Some studies have assessed joint effects of B vitamins. One study assessed pre-pregnancy and third trimester maternal B2, B3, B6, folate and B12 intake, and found a positive correlation between maternal B2 and offspring methylation at *PLAGL1* (*ZAC1*) at both time points.^40^ Another study found no associations between first trimester maternal plasma B12 and B6 concentrations with offspring methylation at *H19, PEG10*/*SGCE* and *PLAGL1*, but there was a positive trend in methylation at *MEG3* across maternal B6 quartiles.^54^

### Choline and betaine

To date there is one human intervention study investigating the effect of supplementing mothers’ diets with choline (480 mg vs 930 mg) in the third trimester on offspring DNA methylation. The intervention increased methylation at *NR3C1* and *CRH* in fetal placental tissue but reduced methylation in cord blood. No effect was seen at *GNAS-AS, IGF2, IL10* or *LEP.*^60^ In observational studies, second trimester choline intake has been inversely associated with *DNMT1* methylation in cord blood.^42^ Third trimester choline intake has been positively associated with *DNMT1* methylation in cord blood and in infant buccal cells.^42^^,^^44^ Maternal periconceptional betaine intake has been positively associated with cord blood methylation at *DNMT1*, *RXRA* and *POMC*,^42–44^ and second trimester intake inversely associated with *LEP* methylation.^42^

### Polyunsaturated fatty acids

Polyunsaturated fatty acids (PUFAs) are thought to influence OCM by upregulating enzymes responsible for the methylation of homocysteine to methionine and by directly influencing demand for methyl groups via phosphatidylcholine (described in Supplementary Material 1, available as Supplementary data at *IJE* online). There have been several studies of PUFA supplementation in mothers. In one trial, omega-3 PUFA supplementation in the second and third trimesters showed no difference in the cord blood methylation of various gene promoter sites, but the intervention increased global methylation (LINE-1) in offspring of mothers who smoked.^69^ It also decreased *H19* methylation, and increased *IGF2* methylation in offspring of overweight mothers.^64^ A more recent trial, also implemented in the second and third trimesters, found omega-3 PUFA supplementation was associated with 21 differentially methylated regions (DMRs) at birth.^65^ These were predominantly hypomethylated in the intervention group. However, not all omega-3 PUFA supplementations trials have demonstrated an effect on methylation.^70^ Maternal plasma omega-6 PUFA concentrations in the third trimester have been inversely associated with offspring *MIRLET7BHG* methylation.^66^

### Broader nutrition measures: famine studies, seasonal exposures, macronutrients

Several studies have used broader measures of maternal nutritional exposure, such as famine, season of conception and macronutrient intake. During the Dutch Famine of 1944, there was a large drop in all food intakes, with average energy intake reduced to 500–1000 kcal per day.^71^ In follow-up studies of adults who were exposed to famine *in utero*, exposure in early pregnancy (periconception and up to 10 weeks of gestation) was associated with lower methylation of *INSIF* and *TMEM105*, increased methylation at *IL10*, *GNASAS*, *LEP*, *ABCA1*, *MEG3*, *TACC1* and *ZNF385A*, and both increased and decreased methylation at *IGF2* depending on the loci within the gene.^45–48^ Not all these effects were seen in those exposed during late gestation.^45^^,^^48^ In a candidate gene analysis of putative metastable epialleles, offspring exposed to famine for at least 7 months during gestation in Bangladesh had higher methylation at *PAX8* and lower methylation at *PRDM9* and *ZFP57*, compared with unexposed controls.^49^

One study found an inverse association between maternal second trimester carbohydrate intake and infant *RXRA* methylation.^55^ Another study looked at the effect of a prenatal diet high in fat and sugar and found a positive association with offspring *IGF2* methylation.^63^ Higher methylation at *GR* has been observed in infants of mothers having higher meat/fish/vegetables and lower bread/potato intake in late pregnancy (>20 weeks of gestation compared with earlier in pregnancy) and increased infant methylation at *HSD2* has been associated with increased maternal meat and fish intake in late pregnancy.^62^ In a pilot trial of periconceptional multiple micronutrient supplementation (UNIMMAP) for mothers, there were sex-specific effects on infant methylation at *IGF2R*, *GNASAS*, *MEG3* and *MEST.*^53^ The difficulty of such studies, however, is that it is not possible to know which nutrient deficits or imbalances caused the epigenetic effects. In The Gambia, where season has marked effects on maternal diet and body weight,^72^ children conceived in the rainy season had higher methylation in peripheral blood lymphocytes at six MEs, at *VTRNA2–1* and at *POMC* compared with those conceived in the dry season.^31^^,^^41^^,^^43^ This may reflect a role of one-carbon-related nutrients; in the rainy season, maternal periconceptional plasma showed higher concentrations of folate, B2, methionine, betaine, S-adenosyl methionine (SAM):S-adenosyl homocysteine (SAH) ratio and betaine:dimethylglycine (DMG) ratio, and lower B12 and homocysteine, indicating higher methylation potential.

Aside from those considered above, the list of maternal exposures associated with changes in infant DNA methylation continues to grow. These include further nutrition-related exposures (e.g. dietary polyphenols,^73^ vitamin D^74^^,^^75^ and vitamin A^76^) non-nutrition-related exposures (e.g. maternal stress^77^ and toxin exposure^78^) and factors that span the spectrum of nutrition and health-related considerations (e.g. maternal hyperglycaemia,^79^ maternal body mass index (BMI),^80–82^ intrauterine growth restriction (IUGR),^83–85^ the microbiome^86^ and infection^87^). The ongoing challenge is not only to identify relevant exposures, but also to delineate the consequences for human health across the life course. It is to this latter point that we now turn.

## Review of studies linking nutrition-associated DNA methylation loci to health outcomes

In animal studies, nutritional exposures in pregnancy bring about distinct phenotypic effects in offspring via epigenetic mechanisms. Differential methylation of genes may induce phenotypic variation by the modulation of gene expression which may alter tissue structure, homeostatic control processes and the activity of metabolic pathways.^88^ Often cited examples include the effects of maternal methyl donor supplementation on offspring coat colour and adiposity in the Agouti mouse, and the development of the fertile queen bee from genetically identical larvae by epigenetic silencing of *DNMT3*, caused by preferential feeding of royal jelly.^9^^,^^89^

In this section we focus on evidence provided by two types of studies:

- Those reporting associations between methylation at the nutrition-sensitive epigenetic loci described above and offspring phenotypes; these are summarized in Table 2, with detailed information on all included studies (*n* = 31) in Supplementary Table 2, available as Supplementary data at *IJE* online;

**Table 2.** Summary of associations between methylation at nutrition-sensitive genetic loci and phenotypes

Direction of DNA methylation/locus	Associated phenotype/direction	Tissue analysed	Age at methylation measurement
	(↑/↓: increased/decreased)		
Birth size			
↑*H19*,^56^*↑PLAGL1*,^56^*↓MEG3*,^56^*↓MIRLET7BHG*,^66^*↑IGF2*^90^	*↑*Birthweight	Cord blood	Birth
*↑IGF2 DMR2*^91^	*↑*Birthweight	Placenta	Birth
*↓IGF2*,^52^*↑HSD2*^62^	*↑*Birthweight	Peripheral blood	17 months,^52^ 40 years^62^
*↑H19* ICR^62^	*↓*Birth length	Peripheral blood	40 years
*↑PLAGL1*^40^	*↑*Estimated fetal weight at 32 weeks of gestation	Cord blood	Birth
*↑HSD2*^62^	*↓*Neonatal ponderal index	Peripheral blood	40 years
*↓IGF2 DMR0*,^83^*↑H19*^92^	*↑*Small for gestational age	Cord blood	Birth
*↑MEST*,^93^*↑LEP*^94^	*↑*Small for gestational age	Placenta,^93^ cord blood^94^	Birth
*↓IGF2 DMR0*^95^	*↑*Small for gestational age	Peripheral blood	11 years
Anthropometric measures/adiposity			
*↑PLAGL1*^40^	*↑*Weight at age 1 year	Cord blood	Birth
*↑PLAGL1*^40^	*↑*Body mass index (BMI) z-score at age 1 year	Cord blood	Birth
*↑IGF2 DMR2*^91^	*↑*Height, head and thorax circumference at birth	Placenta	Birth
*↑POMC*^96^	*↑*Obesity at age 11 years	Peripheral blood	11 years
*↑IGF2/H19* ICR^97^	*↓*Early childhood head circumference	Peripheral blood	1–10 years
*↑H19* ICR,^62^*↑HSD2*^62^	*↑*Weight in adulthood	Peripheral blood	40 years
*↑H19* ICR,^62^*↑HSD2*,^62^*↑NR3C1 exon 1C*^62^	*↑*Waist circumference in adulthood	Peripheral blood	40 years
*↑*POMC,^43^*↑H19* ICR,^62^*↑*HSD2,^62^*↑ NR3C1 exon* 1C,^62^*↓LEP*^98^	*↑*BMI in adulthood	Peripheral blood	48,^43^ 40,^62^ 34.7^98^ years
*↑RXRA*^55^	*↑*Adiposity at age 9 years	Cord blood	Birth
*↓LEP*^99^	*↑*Obesity at age 10–15 years	Saliva	10–15 years
*↓LEP*^100^	*↑*Obese subjects with insulin resistance at age 10-16 years	Peripheral blood	10–16 years
*↑IGF2/H19* ICR^97^	*↑*Skinfold thickness and subcutaneous adiposity at age 17 years	Peripheral blood	17 years
Skeletal growth and bone quality			
*↓RXRA*^75^	*↑*Bone mineral content at age 4 years	Cord blood	Birth
Cardiometabolic outcomes			
*↑LEP*^98^	*↑*Fasting low-density lipoproteincholesterol levels in adulthood	Peripheral blood, Subcutaneous adipose tissue	34.7 years
*↑H19* ICR,^62^*↓ NR3C1 exon* 1F,^62^*↑HSD2*^62^	*↑*Blood pressure in adulthood	Peripheral blood	40 years
*↓LEP*^101^	*↑*High-density lipoprotein (HDL) profile	Peripheral blood	17 months
*↑IGF2*^102^	*↑*Triglycerides (TG), *↑*TG:HDL	Peripheral blood	11.6 years
Cognitive outcomes			
*↑IGF2*^63^	*↑*Early onset conduct problem, attention-deficit/hyperactivity disorder	Cord blood	Birth
*↑NR3C1*,^103^^,^^104^*↓HSD2*^103^^,^^104^	*↑*Risk of being in a poorly regulated neurobehavioural profile	Placenta, Buccal cells	Birth
*↑LEP*^105^	*↑*Lethargy and hypotonicity	Placenta	Birth
- Those linking maternal nutrition exposure, infant DNA methylation and offspring phenotypic effects in a single study (*n* = 8); these are summarized in Table 3.

**Table 3.** Studies linking maternal one-carbon metabolites or broader nutritional exposures to offspring DNA methylation and phenotype

Study	Exposure (exposure timing)	Offspring tissue analysed	Genes analysed	Phenotype investigated	Key findings (↑/↓: increased/decreased, ˜ associated with)
Azzi S *et al.*^40^	Pre-pregnancy BMI, vitamins B2, B3, B6, folate, B12 (3 months before conception and last trimester)	Cord blood	*PLAGL1 (ZAC1)*	Pre- and post-natal growth	↑Pre-pregnancy and last trimester vitamin B2 ˜ ↑*ZAC1* methylation ↑Pre-pregnancy BMI ˜ ↑*ZAC1* methylation ↑*ZAC1* methylation index ˜ ↑estimated fetal weight at 32 weeks of gestation, ↑BMI z-scores at age 1 year
Drake AJ *et al.*^62^	Maternal diet: food group analysis (‘Early’ <20 weeks and ‘late’ >20 weeks of gestation)	Peripheral blood	*IGF2*, *H19* ICR, *HSD2*, *NR3C1*	Birthweight, current height, weight, waist circumference, blood pressure	↑Meat/fish/vegetables and ↓bread/potato intake in late pregnancy ˜ ↑*NR3C1 exon 1F* methylation ↑Meat/fish intake in late pregnancy ˜ ↑*HSD2* methylation ↑*HSD2* methylation ˜ ↓neonatal ponderal index, ↑birthweight, ↑adiposity measures and ↑blood pressure in adulthood (age 40 years) ↑*H19* ICR methylation ˜ ↓birth length, ↑weight, ↑waist circumference, ↑BMI and ↑blood pressure in adulthood ↑*NR3C1 exon 1C* methylation *˜* ↑waist circumference, ↑BMI, ↓blood pressure in adulthood
Godfrey KM *et al.*^55^	Maternal carbohydrate intake (2nd trimester)	Cord blood	*RXRA*, *NOS3*, *SOD1*, *IL8*, *PIK3CD*	Adiposity	↓Maternal carbohydrate intake ˜ ↑*RXRA* methylation ↑*RXRA* methylation ˜ ↑childhood fat mass, ↑% fat mass (at age 9 years)
Hoyo C *et al.*^56^	Maternal erythrocyte folate (1st trimester, median 12 weeks of gestation))	Cord blood	*IGF2*, *H19*, *PEG1/MEST*, *PEG3*, *PLAGL1*, *MEG3-IG*, *PEG10/SGCE*, *NNAT*, *DLK1/MEG3*	Birthweight	↑Folate levels ˜ ↓methylation at *MEG3*, *PLAGL1*, *PEG3* and ↑methylation at *IGF2* ↑Methylation at *H19*, *PEG10/SGCE* and *PLAGL1* and ↓*MEG3* methylation ˜ ↑birthweight *MEG3* methylation ˜ strongest evidence for mediating association between folate and birthweight
Kühnen P *et al.*^43^	Maternal 1-carbon metabolites/season of conception (periconception)	Peripheral blood/MSH-positive neurons	*POMC*	Obesity/BMI	Gambian rainy season of conception and associated 1-carbon metabolites ∼ ↑*POMC* methylation ↑*POMC* methylation ∼ ↑BMI, ↑obesity in children and adults
Lin X *et al.*^66^	Maternal BMI, glucose, plasma fatty acids, plasma vitamin D, serum B12, B6, folate, iron, zinc, magnesium (3rd trimester; 26-28 weeks of gestation)	Cord blood	Epigenome-wide association study	Birthweight, size and adiposity at 4 years	↑Maternal omega-6 PUFA ˜ ↓cg25685359 (*MIRLET7BHG*) methylation ↓ *MIRLET7BHG* methylation ˜ ↑birthweight
Rijlaarsdam J *et al.*^63^	High-fat and -sugar diet (3rd trimester, 32 weeks of gestation)	Cord blood, peripheral blood at age 7 years	*IGF2*	ADHD	Prenatal highfat and high sugar diet ˜ ↑*IGF2* methylation ↑*IGF2* methylation ˜ ↑ADHD symptoms in early-onset persistent conduct (EOP) children age 7 years
Steegers-Theunissen RP *et al.*^52^	Maternal folic acid supplementation (periconception)	Peripheral blood	*IGF2*	Birthweight	↑Folic acid supplementation ˜ ↑*IGF2* methylation at 17 months ↑*IGF2* methylation ˜ ↓birthweight

ADHD, attention-deficit/hyperactivity disorder; BMI, body mass index; ICR, imprinting control region; PUFA, polyunsaturated fatty acids.

We consider three broad categories of offspring phenotypic outcomes: growth and body composition, cardiometabolic risk markers and cognitive function.

### Growth and body composition

DNA methylation signatures in different tissues such as cord and peripheral blood, placenta, subcutaneous and visceral adipose tissue and buccal cells have been associated with growth outcomes such as size at birth (usually birthweight, with or without adjustment for gestational age), child/adult adiposity and skeletal growth or bone size/quality (see Supplementary Table 2, available as Supplementary data at *IJE* online).

*Birth size:* most studies investigating growth-related phenotypes have analysed imprinted genes due to their known role in fetal growth regulation.^106^ Chromosomal region 11p15.5 contains two imprinting control regions (ICRs): the *H19/IGF2* (ICR1) and *KCNQ1/CDKN1C* (ICR2) domains.^107^ Russell–Silver Syndrome (RSS, a disorder of impaired growth) is associated with hypomethylation of ICR1 and hypermethylation of ICR2. Beckwith-Wiedemann Syndrome (BWS, an over-growth disorder) is associated with hypermethylation of ICR1 and hypomethylation of ICR2.^108^ Some studies indicate that patients with RSS and BWS exhibit abnormal methylation at multiple gene loci.^109^ Differences in methylation at these loci have also been associated with less extreme growth-related phenotypes. In a study of 50 French-Canadian mothers and infants, 31% of variance in birthweight was attributed jointly to differential *IGF2/H19* methylation and genotype of a particular *IGF2/H19* polymorphism (rs2107425).^91^ The direction of association between methylation and birthweight, however, varies by study and tissue analysed.^90^^,^^91^ For example, hypomethylation at *IGF2* DMRs have been associated with both increased and decreased birthweight.^52^^,^^83^^,^^90^^,^^95^^,^^110^ Some studies have found no association with birthweight.^111^ Further examples of the complex relationship between DNA methylation at various *IGF2/H19* DMRs and infant growth phenotypes are detailed in Supplementary Table 2, available as Supplementary data at *IJE* online.

The paternally expressed imprinted gene *MEST* acts as an inhibitor of human adipogenesis and is involved in skeletal muscle growth and development.^112^ In placenta, increased methylation at the *MEST* transcription start site is correlated with reduced gene expression and IUGR.^93^^,^^113^ Increased methylation at the paternally expressed *PLAGL1*, which codes for a cell growth suppressor protein, is associated with higher birthweight and weight at 1 year of age.^40^

Some studies have associated other (non-imprinted) genes with birth size. For example, small-for-gestational age newborns had higher methylation at *LEP* in cord blood than appropriate-for-gestational age infants.^94^ Methylation at CpGs within *HSD11B2*, which codes for the enzyme responsible for catalyzing the conversion of cortisol to inactive cortisone, has been inversely related to newborn ponderal index in a cohort study.^62^

A small number of studies have investigated links between maternal nutrition, DNA methylation and newborn size. One study found that higher maternal erythrocyte folate levels in the first trimester were associated with decreased methylation in cord blood at *MEG3*, *PLAGL1* and *PEG3*, and increased methylation at *IGF2.*^56^ Folate concentration and methylation at five DMRs were positively associated with birthweight. The authors hypothesiszed that the association of folate with birthweight could be mediated by differential methylation at *MEG3*, *H19* and *PLAGL1*, with *MEG3* contributing the strongest effect. Another cohort study found that higher maternal plasma glucose and omega-6 PUFA concentrations in the third trimester were associated with increased infant methylation at *IGDCC4* and *CACNA1G*, and decreased methylation at *MIRLET7BHG.* These methylation patterns were all associated with higher birthweight.^66^

*Adiposity:* a case-control study in Germany found that obese adults (BMI >35 kg/m^2^) demonstrated lower methylation at *MEST* than in controls (BMI <25 kg/m^2^), and used a separate dataset to suggest that such outcomes may be partially caused by intrauterine exposure to gestational diabetes mellitus.^114^ In obese boys from the USA, an inverse association was reported between *LEP* methylation in buccal DNA and BMI, waist circumference (as z-scores) and percentage body fat.^99^*NR3C1* Exon 1 C methylation has been positively associated with waist circumference and BMI at age 40 years,^62^ and increased *IGF2/H19* methylation has been associated with increased skinfold thickness and subcutaneous adiposity at age 17 years.^97^

A number of studies have investigated maternal nutritional exposure, DNA methylation and child adiposity. *POMC* codes for melanocyte-stimulating hormone (MSH) and is involved with leptin in the regulation of body weight. *POMC* is an ME, and children conceived in the dry season in The Gambia had lower DNA methylation at a *POMC* variably methylated region (VMR) compared with those conceived in the rainy season.^43^*POMC* VMR methylation influences *POMC* expression,^96^ and methylation at this locus in blood and MSH-positive neurons is associated with BMI and obesity in children and adults.^43^ Godfrey *et al.* (2011) found that lower carbohydrate intake during early pregnancy was associated with increased umbilical cord tissue methylation at *RXRA*, which in turn was associated with greater adiposity in the offspring at 9 years of age.^55^

*Skeletal growth and bone quality*: *RXRA* forms heterodimers with vitamin D (and other nuclear) receptors, facilitating their role in the regulation of bone metabolism.^115^^,^^116^ Differential methylation of specific CpGs in *RXRA* in cord blood DNA has been inversely associated with percentage bone mineral content and bone mineral content adjusted for body size, measured at age 4 years, and also with maternal free 25(OH)-vitamin D index.^75^

### Cardiometabolic outcomes

Maternal nutritional status during pregnancy and factors influencing fetal growth have been implicated in the aetiology of cardiometabolic outcomes such as dyslipidaemia, hypertension, type 2 diabetes (T2D) and cardiovascular disease later in life.^117^^,^^118^

Leptin has been studied extensively in the domain of cardiometabolic outcomes, owing to its role in metabolism and regulation of body weight.^119^*LEP* methylation at a specific CpG in blood and subcutaneous adipose tissue has been positively associated with low-density lipoprotein cholesterol levels in very obese (BMI >40 kg/m^2^) adults.^98^ In the same study, methylation at the *LEP* promoter was inversely correlated with BMI.^98^ A different study found an inverse relationship between *LEP* methylation in whole blood and high-density lipoprotein cholesterol levels in 17-month-old infants.^101^ Furthermore, lower methylation in CpGs near the *LEP* transcription start site has been observed in adolescents with obesity and insulin resistance, although not with obesity alone.^100^*IGF2* methylation has also been related to lipid profile in obese children aged 11 years; those with intermediate methylation at the *IGF2* P3 promoter had higher triglycerides (TG) and a higher TG:high-density lipoprotein cholesterol ratio than those with hypomethylation.^102^*HSD2* methylation has been positively associated with systolic blood pressure,^62^ and *NR3C1* exon1F and *H19* ICR methylation also show positive associations with both systolic and diastolic blood pressures in adults.^62^ Note that adiposity and obesity (reviewed above) are also important risk factors that, alongside other markers, can signal increased risk of adverse cardiometabolic outcomes.^120^

### Cognitive outcomes

The glucocorticoid receptors modulate the action of glucocorticoids and are involved in brain development and function.^121^*NR3C1* and *HSD11B2* genes regulate the action of cortisol and have been well studied in relation to neurobehaviour. Increased methylation at the *NR3C1* promoter and decreased methylation in *HSD11B2* in placental and infant buccal cell DNA have been associated with a high-risk neurobehavioural profile characterized by poor attention, high excitability, low quality of movement and signs of stress.^103^^,^^104^ An increase in *LEP* methylation in placental DNA has been associated with an increased risk of lethargy and hypotonia among male infants.^105^ Increased methylation at *IGF2* in cord blood has been associated with early onset persistent attention-deficit/ hyperactivity disorder (ADHD) in children between 7 and 13 years of age.^63^

## Candidate gene data summary

In Table 4 we provide further details of the 45 ‘candidate genes’ highlighted so far in this review. This includes information on their genomic location, the studies that considered them, regions of interest (ROIs) analysed and the coverage of ROIs on Illumina Infinium Methylation beadchip arrays.

**Table 4.** Candidate genes exhibiting associations between nutritional exposures during periconception and pregnancy and offspring DNA methylation. Links between methylation at nutrition-sensitive genes and offspring phenotype are also included

Gene/region of Interest	Genomic features^c^	Exposure (↑/↓: increased/decreased)	Outcome (↑/↓: increased/decreased)	Coordinates of ROI in studies^d,e^ (number of CpGs on 450k^a^ and EPIC^b^ arrays)
Blue = ME				
Brown = imprinted				
Yellow = ME and imprinted				
*ABCA1* (ATP Binding Cassette Subfamily A Member 1)	Promoter marks; CpG island; binding site for multiple TFs	Famine	↑Methylation^47^	chr9: 107, 690, 502-107, 690, 821 (1)^a^(5)^b^
*ACADM* (Acyl-CoA Dehydrogenase, C-4 To C-12 Straight Chain)	Multiple TFs binding sites; Promoter mark; Active Enhancer mark	↑Folate	↑Methylation^61^	chr1: 76, 189, 707-76, 190, 008 (6)^a^(7)^b^
*BOLA3* (BolA Family Member 3)	Enhancer and Promoter marks; CpG island; binding site for multiple TFs	Rainy season conception	↑Methylation^30^	chr2: chr2: 74, 357, 632-74, 357, 837 (1)^a,b^
*CRH* (Corticotropin-Releasing Hormone)	Enhancer mark	↑Choline	↓Methylation^60^	chr8: 67, 090, 692-67, 091, 132 (5)^a^(8)^b^
*CYS1* (Cystin 1)	Multiple TFs binding sites; Promoter mark	↑Folate	↓Methylation^50^	chr2: 10, 220, 719
*DNMT1* (DNA Methyltransferase 1)	Multiple TFs binding sites; Promoter mark; Active Enhancer mark	↑Folate	↑Methylation^44^, ↓Methylation^42^	chr19: 10, 305, 774-10, 305, 811 (2)^a,b^
	Multiple TFs binding sites; Promoter mark; Active Enhancer mark	↑Folic acid	↓Methylation^44^	chr19: 10, 305, 774-10, 305, 811 (2)^a,b^
	Multiple TFs binding sites; Promoter mark; Active Enhancer mark	↑Choline	↑Methylation^44^, ↑↓Methylation^42^	chr19: 10, 305, 774-10, 305, 811 (2)^a,b^
	Multiple TFs binding sites; Promoter mark; Active Enhancer mark	↑Betaine	↑Methylation^42^	chr19: 10, 305, 774-10, 305, 811 (2)^a,b^
*EXD3 (FLJ20433)* (exonuclease 3'-5' domain containing 3)	Active Enhancer mark; CpG island	Rainy season conception	↑Methylation^30^	chr9: 140, 312, 206-140, 312, 339
*FAM150B* (Family With Sequence Similarity 150, Member B)	None	Famine	↑Methylation^48^	chr2: 366, 113 (1)^a,b ^
*FZD7* (Frizzled Class Receptor 7)	Multiple TFs binding sites; Promoter mark	↑Folate	↑methylation^61^	chr2: 202, 901, 045-202, 901, 470 (5)^a^(4)^b^
*GNASAS* (Guanine Nucleotide Binding Protein (G Protein), Alpha Stimulating Activity Antisense RNA 1)	Enhancer marks; Multiple TFs binding sites	Famine (periconceptional)/Famine (late gestation)	↑Methylation/ ↓Methylation^47^	chr20: 57, 425, 815-57, 426, 108 (3) ^a,b^
	CpG island; MYC binding site	UNIMMAP (supplementation)	↓Methylation^53^	chr20: 57, 429, 802-57, 430, 242 (1)^a^(2)^b^
*H19*	Multiple TFs binding sites	↑Methylation	↑Birthweight^56^	chr11: 2, 011, 131-2, 011, 275 (2)^a,b^
	MYC and CTCF binding sites; Active promoter mark; weak enhancer mark	↑Methylation	↑Small for gestational age^92^	chr11: 2, 019, 727-2, 019, 921 (7)^a^(6)^b^
	Multiple TFs binding sites	↑ Omega-3 PUFA	↓Methylation^64^	chr11: 2, 024, 197-2, 024, 340
	Multiple TFs binding sites	↑Folic acid	↓Methylation^51^	chr11: 2, 024, 254-2, 024, 261
	Enhancer Mark; CTCF-binding site	↑Methylation	↓Birth length, ↑weight in adulthood, ↑adult BMI, ↑adult blood pressure^62^	chr11: 2, 021, 072-2, 021, 291 (2)^a,b^
*HSD11B2* (Hydroxysteroid 11-Beta Dehydrogenase 2) *(HSD2)*	Multiple TFs binding sites; CpG island	↑Methylation	↓Neonatal ponderal index, ↑birthweight, ↑adult adiposity, ↑adult blood pressure^62^	chr16: 67464346-67464649 (3)^a^(4)^b^
	Multiple TFs binding sites; Promoter mark; Active Enhancer mark; CpG island	↑Meat and fish intake	↑Methylation^62^	chr16: 67, 464, 981-67, 465, 111 (1)^a^(2)^b^
	Multiple TFs binding sites, Active Enhancer mark	↓Methylation	↑Risk of being in a poorly regulated neurobehavioral profile^103^^,^^104^	chr16: 67, 464, 387-67, 464, 417
*IGF2* (Insulin-like Growth Factor 2)	POL2A binding site	↑Folic acid	↓Methylation^44^	chr11: 2, 151, 629-2, 151, 721 (3) ^a,b^
	POL2A binding site	↑Folate	↑Methylation^56^	chr11: 2, 151, 629-2, 151, 721 (3) ^a,b^
	1 reported SNP (rs3741210)	↑Omega-3 PUFA	↑Methylation^64^	chr11: 2, 169, 425-2, 169, 556
	CTCF binding site; Enhancer mark; 2 reported SNPs (rs3741210, rs3741208)	↑Folic acid	↑Methylation^52^	chr11: 2, 169, 459 -2, 169, 796
	CTCF binding site; Enhancer mark; 2 reported SNPs (rs3741210, rs3741208)	↑Methylation	↓Birthweight^52^	chr11: 2, 169, 459 -2, 169, 796
	CTCF binding site; Enhancer mark; 2 reported SNPs (rs3741210, rs3741208)	Famine	↓Methylation^45^^,^^46^	chr11: 2, 169, 459-2, 169, 796
	POL2A and USF1 binding sites; 1 CpG island; 1 reported SNP (rs1803647)	↑Folic acid	↑Methylation^58^	chr11: 2, 154, 262-2, 154, 977 (5)^a,b^
	Multiple TFs binding sites; Promoter mark; Active Enhancer mark	↑Methylation	↑ADHD in early‐onset persistent youth^63^	(37)^a^(35)^b,f^
	Multiple TFs binding sites; Promoter mark; Active Enhancer mark	High‐fat and ‐sugar diet	↑Methylation^63^	(37)^a^(35)^b,f^
	POL2A binding site; Promoter mark; Active Enhancer mark; CpG island	↑Omega-3 PUFA	↑Methylation^64^	chr11: 2, 159, 107-2, 159, 965 (3)^a^(4)^b^
	EZH2 and CTCF binding site; Promoter mark; CpG island	↑Vitamin B12	↓Methylation^59^	chr11: 2, 161, 115-2, 161, 275 (4)^a,b^
	CTCF binding site; Enhancer mark; 2 reported SNPs (rs3741210, rs3741208)	Famine	↓Methylation^46^	chr11: 2, 169, 385-2, 169, 489
	Enhancer mark	Famine	↓Methylation^46^	chr11: 2, 170, 541-2, 170, 644
	CTCF binding site; Enhancer mark; 2 reported SNPs (rs3741210, rs3741208)	↓Methylation	↑Small for gestational age^83^	chr11: 2, 169, 458-2, 169, 796
	EZH2, RAD21 and CTCF binding site; Promoter mark; CpG island	Famine	↑Methylation^46^	chr11: 2, 160, 906-2, 161, 372 (14)^a^(13)^b^
	EZH2, ZBTB7A and CTCF binding site; Promoter mark; CpG island	Famine	↑Methylation^46^	chr11: 2, 161, 550-2, 161, 846 (1)^a^(2)^b^
	Enhancer mark; 1 reported SNPs (rs3741210)	↓Methylation	↑Small for gestational age^95^	chr11: 2, 169, 467-2, 169, 640
	POLR2A and ZBTB7A binding site	Famine	↓Methylation^46^	chr11: 2, 155, 447-2, 155, 736 (1)^a,b^
	CpG island; USF1 and POL2A binding sites	↑Methylation	↑Birthweight, birth height, head and thorax circumference at birth^91^	chr11: 2, 154, 263-2, 154, 457 (2)^a,b^
	None	↑Methylation	↑Birthweight^90^	chr11: 2, 169, 518-2, 169, 499
	CTCF and REST binding sites; CpG island	↑Methylation	↑TG and TG: HDL^102^	chr11: 2, 160, 374-2, 160, 610 (4)^a,b^
*IGF2R* (Insulin-like Growth Factor 2 Receptor)	CpG island; associated with SNP rs677882 and rs8191722	↑UNIMMAP (supplementation)	↓Methylation^53^	chr6: 160, 426, 403-160, 426, 850
*IGF2/H19 ICR*	None	↑Methylation	↓Head circumference between 1–10 years; ↑subcutaneous fat measures at age 17 years^97^	chr11: 2, 064, 402-2, 064, 717
*IL10* (Interleukin 10)	Enhancer and Promoter marks; binding site for multiple TFs	Famine	↑Methylation^47^	chr1: 206, 946, 011-206, 946, 339 (2)^a^(3)^b^
*INSIGF* (Insulin- Insulin-like Growth Factor 2)	None	Famine	↓Methylation^46^^,^^47^	chr11: 2, 182, 336-2, 182, 640 (5)^a^(4)^b^
*LASP1* (LIM And SH3 Protein 1)	Multiple TFs binding sites; Promoter marks; Enhancer marks; 4 CpG islands; 25 reported SNPs	↑Folate	↑Methylation^61^	chr17: 37, 123, 638-37, 123, 949 (9)^a,b^
*LEP* (Leptin)	None	↑Folate	↓Methylation^42^^,^^44^	chr7: 127, 881, 035-127, 881, 054
	None	↑Betaine	↓Methylation^42^	chr7: 127, 881, 035-127, 881, 054
	None	↑Folic acid	↑Methylation^42^	chr7: 127, 881, 035-127, 881, 054
	CpG island; CEBP binding site; 2 reported SNPs (rs791620, rs2167270)	Famine	↑Methylation^47^	chr7: 127, 881, 054-127, 881, 410 (4)^a^(6)^b^
	CpG island; CEBP binding site; 2 reported SNPs (rs791620, rs2167270)	↑Methylation	↑Small for gestational age^94^	chr7: 127, 881, 127-127, 881, 350 (4)^a^(6)^b^
	CpG island; 1 reported SNP (rs2167270)	↓Methylation	↑BMI^100^	chr7: 127, 881, 280-127, 881, 300 (2)^a^(3)^b^
	CpG island; CEBP binding site; 2 reported SNPs (rs791620, rs2167270)	↓Methylation	↑BMI; ↑hip circumference^98^	chr7: 127, 881, 126-127, 881, 474 (3)^a^(4)^b^
	CpG island; CEBP binding site; 2 reported SNPs (rs791620, rs2167270)	↑Methylation	↑Fasting LDL-C^98^	chr7: 127, 881, 126-127, 881, 474 (3)^a^(4)^b^
	CpG island	↓Methylation	↑BMI^99^	chr7: 127, 881, 036 -127, 881, 057
	CpG island; CEBP binding site; 2 reported SNPs (rs791620, rs2167270)	↑Methylation	↑Lethargy and hypotonicity^105^	chr7: 127, 881, 127-127, 881, 350 (4)^a^(6)^b^
	CpG island; CEBP binding site; 2 reported SNPs (rs791620, rs2167270)	↓Methylation	↑HDL^101^	chr7: 127, 881, 053-127, 881, 410 (4)^a^(6)^b^
*LY6E* (Lymphocyte Antigen 6 Family Member E)	Multiple TFs binding sites; Promoter mark; Active Enhancer mark	↑Folate	↓Methylation^61^	chr8: 144, 120, 106-144, 120, 706 (8)^a^(9)^b^
*MEG3* (Maternally Expressed 3) *(GTL-2)*	CpG island; Promoter mark	↑Vitamin B6	↑Methylation^54^	chr14: 101, 294, 220-101, 294, 391
	CpG island; Promoter mark	↑Folate	↓Methylation^56^	chr14: 101, 294, 220-101, 294, 391
	Enhancer and Promoter marks; CpG island; *POLR2A* binding site	↑ UNIMMAP (supplementation)	↓Methylation^53^	chr14: 101, 292, 283– 101, 292, 796 (4)^a^(5)^b^
	CpG island; Promoter mark	↓Methylation	↑Birthweight^56^	chr14: 101, 294, 220-101, 294, 391
	None	Famine	↑Methylation^47^	chr14: 101, 291, 413-101, 291, 642 (5)^a^(6)^b^
*MEST* (Mesoderm-Specific Transcript) *(PEG1)*	CpG island	↑UNIMMAP (supplementation)	↓Methylation^53^	chr7: 130, 131, 325-130, 131, 792 (11)^a^(9)^b^
	Multiple TFs binding sites; Promoter mark; Enhancer mark; CpG island	↑Methylation	↑Small for gestational age^93^	chr7: 130, 125, 200-130, 126, 400 (16)^a^(17)^b^
*MIRLET7BHG* (MicroRNA Let-7b Host Gene)	Active Enhancer mark	↑Omega-6 PUFA	↓Methylation^66^	chr22: 46, 473, 721 (1)^a,b ^
	Active Enhancer mark	↓Methylation	↑Birthweight^66^	chr22: 46, 473, 721 (1)^a,b ^
*NR3C1* (Nuclear Receptor Subfamily 3 Group C Member 1) (*GR*)	Multiple TFs binding sites; Promoter mark; Enhancer mark; CpG island; 2 reported SNPs (rs10482604, rs10482605)	↑Methylation	↑Risk of being in a poorly regulated neurobehavioural profile^103^^,^^104^	chr5: 142, 783, 501-142, 783, 640 (4)^a,b^
	Multiple TFs binding sites; Promoter mark; Enhancer mark; CpG island; 2 reported SNPs (rs10482604, rs10482605)	↑Choline	↑Methylation^60^	chr5: 142, 783, 501-142, 783, 908 (5)^a^(7)^b^
	Multiple TFs binding sites; Promoter mark; Enhancer mark; CpG island	↑Methylation	↑Adult waist circumference, ↑adult BMI^62^	chr5: 142, 782, 759-142, 783, 164 (2)^ab^
	Multiple TFs binding sites; Promoter mark; Enhancer mark; CpG island; 1 reported SNP (rs10482604)	↑Meat/fish and vegetable intake, ↓bread/potato intake in late pregnancy	↑Methylation^62^	chr5: 142, 783, 579-142, 783, 714 (3)^a,b^
	Multiple TFs binding sites; Promoter mark; Enhancer mark; CpG island; 1 reported SNP (rs10482604)	↑Methylation	↓Adult blood pressure^62^	chr5: 142, 783, 578 -142, 783, 714 (3)^a,b^
*OSBPL5/MRGPRG* (Oxysterol-Binding Protein Like 5/MAS Related GPR Family Member G)	Enhancer mark; CpG island	Famine	↓Methylation^48^	chr11: 3, 225, 076 (1)^a,b ^
*OTX2* (Orthodenticle Homeobox 2)	CpG island; EZH2 binding site	↑Folate	↓Methylation^50^	chr14: 57, 278, 729 (1)^a,b ^
*PAX8* (Paired Box^8^)	Multiple TFs binding sites; Promoter mark; Active Enhancer mark	Rainy season conception	↑Methylation^30^	chr2: 113, 993, 262-113, 993, 391(2)^a,b^
				chr2: 113, 992, 866-113, 993, 036(2)^a,b^
	Multiple TFs binding sites; Promoter mark; Active enhancer mark	Famine	↑Methylation^49^	chr2: 113, 992, 762-113, 993, 313 (8)^a^(7)^b^
*PEG3* (Paternally Expressed 3)	Multiple TFs binding sites; 2 CpG islands; 1 reported SNP (rs2302376)	↑Folate	↓Methylation^56^	chr19: 57, 351, 945-57, 352, 096 (4)^a^(3)^b^
	Multiple TFs binding sites; 2 CpG islands; 1 reported SNP (rs2302376)	↑Folic acid	↓Methylation^56^	chr19: 57, 351, 945-57, 352, 096 (4)^a^(3)^b^
	Multiple TFs binding sites; 2 CpG islands; 1 reported SNP (rs2302376)	↑Folic acid	↓Methylation^58^	chr19: 57, 351, 944-57, 352, 096 (4)^a^(3)^b^
*PLAGL1* (PLAG1-Like Zinc Finger 1) *(ZAC1)*	Multiple TFs binding sites; Promoter mark; Active Enhancer mark; CpG island	↑Folate	↓Methylation^56^	chr6: 144, 329, 109-144, 329, 231 (1)^a,b^
	Multiple TFs binding sites; Promoter mark; Active Enhancer mark; CpG island	↑Methylation	↑Birthweight^56^	chr6: 144, 329, 109-144, 329, 231 (1)^a,b^
	Multiple TFs binding sites; Promoter mark; CpG island	↑Methylation index	↑Fetal weight at 32 weeks of gestation, weight and BMI at 1 year^40^	chr6: 144, 329, 390-144, 329, 740 (4)^a,b^
	Multiple TFs binding sites; Promoter mark; CpG island	↑ Vitamin B2	↑Methylation index^40^	chr6: 144, 329, 390-144, 329, 740 (4)^a,b^
*POMC* (Proopiomelanocortin)	Multiple TFs binding sites; Promoter mark; Active Enhancer mark; CpG island	↑Methylation	↑BMI^43^^,^^96^	chr2: 25, 384, 508-25, 384, 832 (3)^a,b^
	Multiple TFs binding sites; Promoter mark; Active Enhancer mark; CpG island	↑SAM:SAH ratio; ↑betaine	↑Methylation^43^	chr2: 25, 384, 508-25, 384, 832 (3)^a,b^
*PPAP2C (PLPP2)* (Phosphatidic Acid Phosphatase 2c)	CpG island	Famine	↑Methylation^48^	chr19: 292, 167 (1)^a,b ^
*PRDM9* (PR-Domain Containing Protein 9)	Multiple transcription factor binding sites; Promoter mark, Active enhancer mark; 2 reported SNPs (rs10077095, rs1994929)	Famine	↓Methylation^49^	chr5: 23, 507, 030-23, 507, 752 (12)^a^(11)^b^
*RBM46* (RNA-Binding Motif Protein 46)	CpG island	Rainy season conception	↑Methylation^31^	chr4: 155, 702, 818-155, 703, 110 (1)^a,b^
*RXRA* (Retinoid X Receptor Alpha)	Multiple TFs binding sites; Enhancer mark	↑Methylation	↑Fat mass; % fat mass^55^	chr9: 137, 215, 697 -137, 216, 117 (1)^a,b^
	Multiple TFs binding sites; Enhancer mark	↑Methylation	↑BMI^55^	chr9: 137, 215, 697 -137, 216, 117 (1)^a,b^
	Multiple TFs binding sites; Enhancer mark	↑Carbohydrate intake	↓Methylation^55^	chr9: 137, 215, 697 -137, 216, 117 (1)^a,b^
	Multiple TFs binding sites; Enhancer mark	↑Methylation	↓Bone mineral content; % BMC^75^	chr9: 137, 215, 697 -137, 216, 117 (1)^a,b^
	Multiple TFs binding sites; Promoter mark; Active Enhancer mark; CpG island	↑Folate	↓Methylation^42^	chr9: 137, 217, 097-137, 217, 132
	Multiple TFs binding sites; Promoter mark; Active Enhancer mark; CpG island	↑Folate	↑Methylation^44^	chr9: 137, 217, 097-137, 217, 132
*SLC38A2* (Solute Carrier Family 38 Member 2)	Enhancer mark	Famine	↑Methylation^48^	chr12: 46, 737, 123 (1)^a,b ^
*SLITRK1* (SLIT And NTRK-like Family Member 1)	Promoter mark; Enhancer mark; CpG island	Rainy season conception	↑Methylation^30^	chr13: 84, 453, 741-84, 453, 828
				chr13: 84, 454, 210-84, 454, 281
*SPATC1L (C21orf56)* (Spermatogenesis And Centriole Associated 1 Like)	Multiple TFs binding sites; Promoter mark; Active Enhancer mark	↑Folate	↓Methylation^61^	chr21: 47, 604, 052-47, 604, 654 (5)^a,b^
*STX11* (Syntaxin 11)	Multiple TFs binding sites; Promoter mark; CpG island	↑Folate	↓Methylation^50^	chr6: 144, 471, 564 (1)^a,b ^
*TACC1* (Transforming Acidic Coiled-Coil Containing Protein 1)	Promoter mark; Enhancer mark	Famine	↑Methylation^48^	chr8: 38, 586, 183 (1)^a,b ^
*TFAP2A* (Transcription Factor AP-2 Alpha)	E2F1 and EZH2 binding site; Promoter mark; Active Enhancer mark; CpG island	↑Folate	↓Methylation^50^	chr6: 10, 411, 911 (1)^a,b ^
*TMEM105* (Transmembrane Protein 105)	Enhancer mark; Active Enhancer mark; CpG island	Famine	↓Methylation^48^	chr17: 79, 283, 915 (1)^a,b ^
*VTRNA2-1* (Vault RNA 2-1)	Multiple TFs binding sites; Promoter mark; Active Enhancer mark; CpG island	Rainy Season; ↑vitamin B2; ↑methionine; ↓dimethylglycine	↑Methylation^41^	chr5: 135, 415, 762-135, 416, 613 (15)^a^(13)^b^
*WNT9A* (Wnt Family Member 9A)	NRF1 binding site; Promoter mark; Active Enhancer mark; CpG island	↑Folate	↑Methylation^61^	chr1: 228, 075, 423-228, 075, 749 (5)^a^(3)^b^
*ZFP57* (Zinc Finger Protein 57)	YY1 binding site; Promoter mark; Active Enhancer mark; multiple reported SNPs	↑Folate	↓Methylation^61^	chr6: 29, 648, 161-29, 649, 084 (24)^a^(25)^b^
	Promoter mark; Active Enhancer mark; multiple reported SNPs	Famine	↓Methylation^49^	chr6: 29, 648, 345-29, 649, 024 (19)^a^(18)^b^
*ZFYVE28* (Zinc Finger FYVE-Type Containing 28)	Multiple TFs binding sites; Promoter mark; CpG island	Rainy season conception	↑Methylation^30^	chr4: 2, 366, 658-2, 366, 739 (1)^a,b^
				chr4: 2, 366, 909-2, 367, 003
*ZNF385A* (Zinc Finger Protein 385A)	Multiple TFs binding sites; Promoter mark; CpG island	Famine	↑Methylation^48^	chr12: 54, 764, 265 (1)^a,b ^

LBW, low birthweight; LDL-C, low-density lipoprotein cholesterol; ME, metastable epiallele; ROI, region of interest; SAH, s-adenosyl homocysteine; SAM, s-adenosyl methionine; UNIMMAP, United Nations International Multiple Micronutrient Preparation.

a Number of CpGs covered on Infinium HumanMethylation450K BeadChip array.

b Number of CpGs covered on Infinium MethylationEPIC array.

c The following regulatory features were checked: enhancer/promoter marks (histone), overlapping binding sites for various transcription factors (e.g. CTCF, POL2A etc.) within region of interest (ROI) and presence of nearby reported GWAS single nucleotide polymorphisms (SNPs).

d Coordinates based on genome build hg19. The BiSearch Web server^122^ was used to find genomic coordinates for ROIs where only primers were available.

e HumanMethylation450 v1.2 and Infinium MethylationEPIC v1.0 B4 Manifest Files were referred to report ROI coverage on Illumina Infinium Methylation BeadChip arrays.

f A total of 37 probes from 450k array were found within the gene and considered for analysis.

## Discussion

In this review we have described evidence in humans linking maternal nutrition during pregnancy with DNA methylation in the offspring, and linking DNA methylation at nutrition-sensitive loci to phenotypes at birth and outcomes in later life. As with all reviews, publication bias can mean that null findings may have been under-reported, and studies that do report associations may sometimes rely on *post hoc* subgroup analyses for significant findings. There are also numerous challenges specific to both the design and interpretation of intergenerational nutritional epigenetics studies which we discuss in the following sections.

### Measuring nutritional exposures

Methods for measuring maternal nutritional exposure have limitations. For example, one of the most commonly used methods for this purpose are food frequency questionnaires, which suffer from recall bias and have differing validity by micronutrient.^123^ Weighed records require accurate, context-specific dietary databases and well-trained data collectors, and may not accurately reflect normal eating habits.^124^ However, these two approaches have the advantage of capturing food groups and combinations of nutrients that more direct tissue nutritional biomarkers can overlook.^125^ Plasma biomarkers are challenging to interpret, given that they represent nutrient levels after absorption and through interaction with genotype, and are not simple reflections of dietary intake. Concentrations do not capture metabolite flux, and can be misleadingly low if tissue uptake is rapid. Of particular relevance to maternal gestational samples is the effect of haemodilution, which can lower several biomarker concentrations.^126^ Maternal plasma nutrient concentrations are assumed to reflect dietary intake, and to correlate with cord blood concentrations and nutrient levels in fetal tissue, which may not be the case. Whereas positive correlations between maternal serum and cord blood serum are found for homocysteine, betaine, folate and B12, cord blood levels are multiple times higher, suggesting that these nutrients are homeostatically controlled to ensure fetal supply.^127^ In the context of periconceptional studies, more research is needed on which accessible tissues best represent the nutritional milieu surrounding the developing embryo in the initial days after fertilization. In the meantime, serum or plasma levels, though imperfect, are likely to offer a more accurate representation of fetal nutrient exposure than dietary intake methods.

Most of the attention on nutritional exposures has focused on the provision of methyl groups and the necessary co-factors for DNA methylation. However, the periconceptional period is marked by an initial wave of demethylation to erase parental epigenetic marks, before the process of remethylation.^27^ It is therefore important to consider the role nutrition could play in influencing demethylation. In demethylation, 5-methylcytosine is sequentially oxidized to 5-hydroxymethylcytosine and 5-formylcytosine (5fC) by 10-11 translocation (TET) dioxygenases that use vitamin C (ascorbate) as a co-factor.^128^ 5fC can then either be further oxidized to 5-carboxylcytosine or converted to an unmethylated cytosine by base excision repair. Adding vitamin C to mouse or human embryonic stem cells *in vitro* increases the activity of TET enzymes, resulting in active demethylation in the germline.^129^ However, to our knowledge there have been no human *in vivo* studies exploring effects of periconceptional vitamin C deficiency on offspring DNA methylation.

Nutritional compounds do not act in isolation, and ideally analyses should recognize this by considering their interactions in metabolic pathways. For example, one-carbon metabolism is governed by intricately controlled feedback loops which help protect the flux of metabolites, through key reactions over a range of nutrient and co-factor concentrations.^130^^,^^131^ This means that associations between individual micronutrients and methylation (e.g. the commonly analysed methyl donors folate and betaine) can disappear after adjustment for other metabolites (e.g. SAM and DMG, which can inhibit transmethylation reaction rates). Advances in measurement technology that allow the measurement of a greater range of nutritional biomarkers (e.g. metabolomics), combined with more sophisticated analytical techniques,^132^^,^^133^ should enable a more nuanced understanding of the ways in which nutritional biomarkers combine to jointly influence methylation.

### Measuring DNA methylation

A single CpG site in a single cell is either methylated or unmethylated, but measurements are typically made at the tissue level where methylation is a quantitative measure corresponding to the proportion of methylated cells.^134^ Accurate assessment of tissue-level DNA methylation patterns presents a challenge, given the sensitivity of the measurements to both technical and biological variation. The advent of high-throughput, genome-wide microarray platforms, such as the Illumina HumanMethylation 450 K and EPIC arrays,^135–137^ has helped in this regard, first by helping to standardize aspects of epigenome-wide association study (EWAS) design, and second by reducing the cost of genome-wide methylation assays required for adequately powered large studies.

Microarray-based EWAS have a number of limitations. First, by design, only a small proportion of the methylome is interrogated. These platforms attempt to include CpGs sites from all annotated genes, but the number of CpG sites per gene is low and equal coverage is typically not given to all genomic features and/or CpG contexts, with the focus having traditionally been on sites in promoters and CpG islands. Second, arrays provide no information on sequence-level variation, which is known to influence methylation status.^138^^,^^139^ Finally, bioinformatics and analytical expertise are required (as well as the necessary computational resources) to process and model the data, and to correct for batch and other technical effects, in order to obtain reliable, high-quality methylation profiles.^140^ As an alternative, true genome-wide approaches such as whole-genome bisulphite sequencing (WGBS) are available which interrogate all ∼28 million CpG sites in the methylome, although this is currently prohibitively expensive for larger samples. Targeted high-resolution platforms^141^^,^^142^ offer a potential compromise between coverage and cost, but their utility, convenience and cost-effectiveness for performing EWAS remain to be established. Given the importance of demethylation during periconceptional epigenetic remodelling, it may also be important to consider the oxidized forms of 5-methyl cytosine (e.g. 5-hydroxymethylcytosine) which occur as intermediate products in the demethylation pathway.^143^

### Tissue specificity, confounding and stability of methylation across the life course

The tissue-specific nature of DNA methylation presents a major challenge for epigenetic association studies.^134^^,^^144^ The majority of studies reported in this review are constrained to accessible tissues such as cord blood that may be unrelated to the phenotype of interest, and different tissues may be sensitive to different environmental exposures. In this case reference epigenomes from different tissues and cell types in both healthy and diseased individuals^145^ may inform the choice of tissue as well as providing data for investigating the tissue specificity of identified signals. Where exposure-related effects occur during early embryonic development, before gastrulation, methylation changes may be concordant across multiple tissues,^146^ so that methylation states in accessible tissues such as blood and buccal cells may serve as a proxy for methylation in the target tissue.

Furthermore, numerous biological factors may act as potential confounders, for example age, sex, smoking status and BMI. Tissue-specific methylation differences arising from cell type heterogeneity, notably in blood, can also act as confounders,^147^ although there are well-established methods that can be used to correct for this.^147^^,^^148^

DNA sequence polymorphisms are also known to influence DNA methylation status and may confound observed associations.^149^ Heritability of DNA methylation is estimated to be in the range of 18% to 37%.^150^^,^^151^ Consistent with this, many studies have shown that methylation quantitative trait loci (mQTL)—genetic variants associated with methylation differences at the population level—are widespread. To account for this, ideally high-throughput genotype data on the sample being studied should be used^149^ but, if such data are unavailable, population-level reference mQTL data can be informative.^139^

Finally, methylation changes associated with an early-life exposure may change throughout the life course, with implications for their utility as biomarkers of exposure or predictors of later phenotype.^152–154^ Depending on the research question, this may suggest the need to assess long-term stability of methylation at specific loci, through the collection of longitudinal samples.

### Linking methylation changes to gene function

Many of the DNA methylation changes reported in studies covered in this review are small, often within the margins of error of the measuring technology, making it difficult to draw conclusions on their functional relevance.^155^ Indeed, relatively few methylation studies measure gene expression. The link between DNA methylation and expression is complex, depending on genomic context (e.g. location with gene bodies, promoters and enhancers).^156^ This could in part explain seemingly contradictory findings from different studies measuring associations at the same gene. Moreover, a change in methylation may influence transcription factor binding and the induction of a specific signalling pathway in order to observe a change in gene expression. To aid further understanding, future studies should therefore consider measuring transcription factor binding, markers of gene transcription (mRNA levels), and/or translation (protein levels), to better map the potential effects of DNA methylation differences on gene function.^157^

### Capturing phenotypes

In this review we have focused on phenotypic outcomes most commonly considered in the DOHaD context. However, we do not wish to exclude the possibility that there may be a broader range of phenotypes that are implicated. For example, exposure to the Dutch Hunger Winter famine during pregnancy has been associated with a wide variety of offspring phenotypes, varying according to the timing of famine exposure during gestation.^45^^,^^47^ Consideration of the ‘thrifty epigenotype’ hypothesis^24^ would suggest that famine-imposed epigenetic modifications in early life are adaptive where similar environment conditions persist, but maladaptive otherwise. There could therefore be a spectrum of phenotypes according to how great the mismatch is between *in utero* and later life environments. In the case of complex traits such as obesity, the resultant phenotype may also be influenced by factors such as diet and lifestyle in conjunction with methylation differences and genotype of the individual.^158^

### Causal inference

A major goal of nutritional epigenetic studies, also covered in this review, is to assess the potential for epigenetic marks to mediate links between nutritional exposures and health outcomes. In this context, the use of prospective study designs with randomization including negative controls, and techniques such as mediation analysis based on regression systems,^159^ structural equation modelling^160^ or network-based techniques,^161^ parametric/semi-parametric methods,^162^ or instrumental variable approaches such as Mendelian randomization,^80^^,^^163^^,^^164^ can help to strengthen causal inference. More broadly, triangulating findings from diverse studies, each with their own strengths, limitations, assumptions and opposing biases, will maximize the potential for robust findings.^165^^,^^166^

### Study design considerations

The literature in this area is dominated by observational studies. This increases the risk of spurious associations due to confounding or reverse causation,^149^ the latter being a particular problem with methylation association studies where the direction of causality can be hard to establish. Added to this, effect sizes are generally modest, with group-level differences in mean methylation typically less than 10% and often in the region of 1–5% for many of the exposures and phenotypes studied.^155^^,^^167^^,^^168^ This has implications for the design of studies characterizing genome-wide, population-level methylation differences, as they need to be adequately powered to detect potentially small effects after adjusting for multiple testing.^169^

Current interest in periconceptional nutrition has stimulated a number of preconceptional nutrition trials.^170–174^ In these studies, supplementation before conception is necessary to ensure that the conception period is covered and that a maximal effect on maternal nutritional status at conception is achieved. Nonetheless, accurately pinpointing the timing of nutritional exposures to conception is challenging.

## Conclusions

The body of evidence linking maternal nutritional exposure to offspring phenotype via DNA methylation in humans is rapidly growing yet currently remains complex and inconsistent. It is characterized by heterogeneous exposures and outcomes, and mainly observational associations that are frequently under-powered. Existing evidence suggests that the effect of nutritional exposures on DNA methylation depends on the form of the nutritional component, the timing of exposure during periconception and pregnancy, the underlying nutritional status of the mother, maternal and offspring genotype and the specific loci under investigation. The picture is more complex than methylation being determined simply by availability of methyl donors. Many studies have investigated imprinted genes as priority loci for their vulnerability to nutritional exposures, but with the adoption of microarray-based platforms, other candidate genes and gene classes are emerging, for example metastable epialleles.

The utility of this emerging evidence in terms of its translation into effective interventions and therapies remains an open question. Epigenetic marks like DNA methylation may act as integrators of multiple exposures and genetic risk factors, as well as molecular mediators of the effect of exposures on phenotype. Where robust associations are established, DNA methylation can serve as a proxy measure or biomarker of earlier nutritional exposures.^175^ As mediators of the effect on later phenotype, nutritionally sensitive DNA methylation changes can provide a means to identify genes and pathways for targeted interventions. Whereas there is still much work to do in this area, there are grounds for optimism that epigenomic approaches will provide insights into the molecular basis of the developmental origins of health and disease, which could in turn lead to the development of next-generation interventions.

## Funding

This work was supported by the Newton Fund initiative, jointly funded by the Medical Research Council [MR/N006208/1], the Department for International Development, UK, and the Department of Biotechnology, Ministry of Science and Technology, Government of India [BT/IN/DBT-MRC/ DFID/24/GRC/2015–16]. The funding bodies played no role in the design of the review, data collection, analysis, interpretation of data or writing the manuscript.

## The EMPHASIS study group includes

Lena Acolatse, MRC Unit The Gambia at the London School of Hygiene and Tropical Medicine, The Gambia, Meraj Ahmed, Genomic Research on Complex diseases (GRC Group), CSIR-Centre for Cellular and Molecular Biology, Hyderabad, India, Modupeh Betts, MRC Unit The Gambia at the London School of Hygiene and Tropical Medicine, The Gambia, Giriraj R Chandak, CSIR-Centre for Cellular and Molecular Biology, Hyderabad, India, Harsha Chopra, Centre for the Study of Social Change, Mumbai, India, Cyrus Cooper, MRC Life Course Epidemiology Unit, University of Southampton, UK, Momodou K Darboe, MRC Unit The Gambia at the London School of Hygiene and Tropical Medicine, The Gambia, Chiara Di Gravio, MRC Life Course Epidemiology Unit, University of Southampton, UK, Caroline HD Fall, MRC Life Course Epidemiology Unit, University of Southampton, UK, Meera Gandhi, Centre for the Study of Social Change, Mumbai, India, Gail R Goldberg, MRC Elsie Widdowson Laboratory, Cambridge, UK, Prachand Issarapu, Genomic Research on Complex diseases (GRC Group), CSIR-Centre for Cellular and Molecular Biology, Hyderabad, India, Philip James, MRC Unit The Gambia at the London School of Hygiene and Tropical Medicine, UK, Ramatoulie Janha, MRC Unit The Gambia at the London School of Hygiene and Tropical Medicine, The Gambia, Landing M A Jarjou, MRC Unit The Gambia at the London School of Hygiene and Tropical Medicine, The Gambia, Lovejeet Kaur, Genomic Research on Complex diseases (GRC Group), CSIR-Centre for Cellular and Molecular Biology, Hyderabad, India, Sarah H Kehoe, MRC Life Course Epidemiology Unit, University of Southampton, UK, Kalyanaraman Kumaran, MRC Life Course Epidemiology Unit, University of Southampton, UK and CSI Holdsworth Memorial Hospital, Mysore, India, Karen A Lillycrop, University of Southampton, UK, Mohammed Ngum, MRC Unit The Gambia at the London School of Hygiene and Tropical Medicine, The Gambia, Suraj S Nongmaithem, Genomic Research on Complex diseases (GRC Group), CSIR-Centre for Cellular and Molecular Biology, Hyderabad, India, Stephen Owens, Institute of Health and Society, Newcastle University, UK, Ramesh D Potdar, Centre for the Study of Social Change, Mumbai, India, Andrew M Prentice, MRC Unit The Gambia at the London School of Hygiene and Tropical Medicine, The Gambia, Ann Prentice, MRC Unit The Gambia, Elsie Widdowson Laboratory, Cambridge, UK and MRC Life Course Epidemiology Unit, University of Southampton, UK, Tallapragada Divya Sri Priyanka, Genomic Research on Complex diseases (GRC Group), CSIR-Centre for Cellular and Molecular Biology, Hyderabad, India, Ayden Saffari, MRC Unit The Gambia at the London School of Hygiene and Tropical Medicine, UK, Sirazul Ameen Sahariah, Centre for the Study of Social Change, Mumbai, India, Sara Sajjadi, Genomic Research on Complex diseases (GRC Group), CSIR-Centre for Cellular and Molecular Biology, Hyderabad, India, Harshad Sane, Centre for the Study of Social Change, Mumbai, India, Smeeta Shrestha, Genomic Research on Complex diseases (GRC Group), CSIR-Centre for Cellular and Molecular Biology, Hyderabad, India, Matt J Silver, MRC Unit The Gambia at the London School of Hygiene and Tropical Medicine, UK, Ashutosh Singh Tomar, Genomic Research on Complex diseases (GRC Group), CSIR-Centre for Cellular and Molecular Biology, Hyderabad, India, Kate A Ward, MRC Elsie Widdowson Laboratory, Cambridge and MRC Life course Epidemiology Unit, University of Southampton, UK, Dilip Kumar Yadav, Genomic Research on Complex diseases (GRC Group), CSIR-Centre for Cellular and Molecular Biology, Hyderabad, India, Chittaranjan S Yajnik, Diabetes Unit, KEM Hospital and Research Centre, Pune, India.

**Conflict of interest:** None declared. 

## Supplementary Material

Supplementary Material 1

Supplementary Material 2

Supplementary Table 1

Supplementary Table 2
