# Supplementary material for: Candidate genes linking maternal nutrient exposure to offspring health via DNA methylation: a review of existing evidence in humans with specific focus on one-carbon metabolism
Source: Int J Epidemiol. 2018 Aug 17;47(6):1910–37. doi: 10.1093/ije/dyy153 (PMC6280938; doi:10.1093/ije/dyy153)
Supplement: Supplementary Material 1 [file dyy153_supplementary_material_1.pdf]

Supplementary Material 1: Additional information on the role of nutritional factors within one-carbon metabolism

An overview of one-carbon metabolism

S-adenosyl methionine (SAM) is the methyl donor for hundreds of transmethylation reactions[1], and its importance in epigenetics stems from its ability to methylate cytosine bases and histones. The loss of a methyl group from SAM forms S-adenosyl homocysteine (SAH), a build-up of which can reduce methylation rates through allosteric inhibition[2]. The SAM:SAH ratio can be used as a proxy indicator of methylation potential[3]. SAH is further hydrolysed to homocysteine (Hcy)[4] and, since this reaction is reversible, Hcy needs to be removed from the system to maintain favourable methylation conditions. Hcy can be methylated to form methionine or be irreversibly degraded in the transsulfuration pathway. In the case of methionine formation, Hcy can either receive the methyl group from N^5-methyl tetrahydrofolate ('methyl-THF'), produced through the reduction of dietary folates and folic acid, or from betaine in the liver and kidneys, a product formed through the oxidation of choline. Methionine can then be condensed with ATP to form SAM and complete the cycle. Genetic variants in one-carbon enzymes can also affect the flow of metabolites through these pathways[5].

The role of individual nutrients

Folate: Dietary folates and folic acid are reduced to form tetrahydrofolate (THF), which is a scaffold upon which one-carbon units can be attached and activated. THF in turn is reduced to methylene-THF, then to 5-methyl-THF, which donates its methyl group to homocysteine using vitamin B12[6].

B vitamins: Several B vitamins act as cofactors in one-carbon pathways (B12, B2, B6). Vitamin B12 is a cofactor in the methylation of homocysteine by 5-methyl-THF. Vitamin B6 (in the active form of pyridoxal-5'-phosphate; PLP) is required to reduce THF to methylene-THF, and is also a cofactor in the transsulfuration pathway converting homocysteine to cysteine[7]. B2 is required as a precursor to FAD, which is a cofactor for MTHFR to reduce methylene-THF to methyl-THF[3]. The different forms of THF are interconvertible, except the methyl-THF form. In the absence of sufficient B12 to utilise the methyl group from methyl-THF in homocysteine methylation (a process which re-generates THF), methyl-THF can accumulate at the expense of other THF forms. This is termed the 'folate trap' or 'methyl trap' and means pathways dependent on other forms of THF (e.g. purine synthesis and the thymidylate pathway) can become impaired[8].

Choline can be synthesised endogenously or obtained from the diet (good sources include red meat, poultry, milk, eggs and fish[9]). A two-step oxidation reaction converts it to **betaine**[10], which can donate its methyl group to homocysteine via betaine-homocysteine methyl transferase (BHMT)[10]. Betaine can also be directly sourced from the diet (e.g. wheat bran, wheat germ, spinach, beets[9]).

Polyunsaturated fatty acids (PUFAs) influence the 1-carbon pathway through two mechanisms. Firstly, ω-3 PUFAs upregulate enzymes responsible for the methylation of homocysteine to methionine[11,12]. Secondly, the availability of PUFAs can influence methyl balance via phosphatidylcholine (PC). PC is used in the production of very low density lipoproteins (VLDLPs), bile and surfactants. It is formed via two main pathways: the cytidine diphosphocholine (CDP)–choline ('Kennedy') pathway and the PEMT pathway, which converts phosphatidylethanolamine (PE) to PC (Fig. 1). PC differs in composition according to the pathway of its formation; the former incorporates more saturated fatty acids and the latter more PUFAs, such as arachidonic acid (AA) and

docosahexaenoic acid (DHA)[13]. In the PEMT pathway the conversion of a molecule of PE to PC requires three methyl groups from SAM. In rodent studies imbalances in methyl donor supply and the coenzymes involved in 1-carbon metabolism affect PEMT activity, manifested in disturbances to tissue levels of PUFAs[12,14], since the PEMT pathway is the major way phospholipids are transported from the liver to the plasma and other tissues[15,16]. These effects have been most pronounced in pregnant dams consuming a diets designed to resemble a rural Indian intake, with excess folic acid and restricted vitamin B12 lowering offspring placental and brain DHA, as well as lowering placental global methylation[17,18]. In these experiments, supplementation with ω-3 PUFAs reversed the effect of the micronutrient imbalances[12]. The PEMT pathway represents a major demand for methyl groups[19,20]. Hence any factor that reduces the demand for methyl groups in the PEMT pathway (e.g. low PUFA intake) could in theory make more methyl groups available for DNA methylation, with potential consequences for epigenetic outcomes[21].

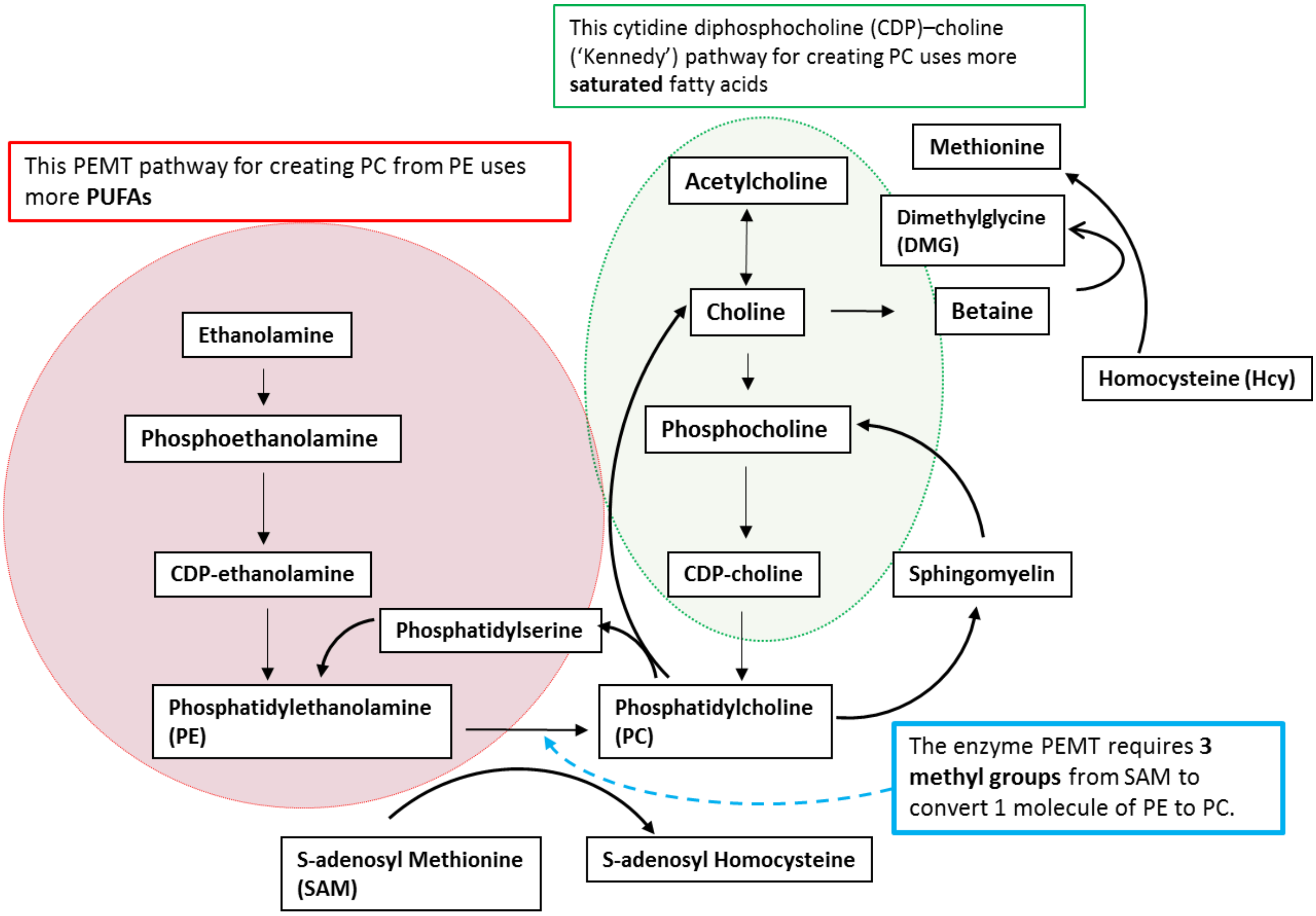

Fig 1: Formation of phosphatidylcholine by two metabolic pathways

Abbreviations: CDP, cytidine diphosphocholine; PC, phosphatidylcholine; PE, phosphatidylethanolamine; PEMT, phosphatidylethanolamine N-methyltransferase; PUFA, polyunsaturated fatty acids; SAM, S-adenosyl methionine.

Amino acids: Several amino acids are thought to contribute 1-carbon units[22], and in turn deficiencies in 1-carbon-related analytes may affect amino acid metabolism[23–25]. The most documented amino acids in this context are serine and glycine. Serine donates a 1-carbon unit at the stage of converting THF to methylene-THF through the action of Serine Hydroxymethyltransferase (SHMT) and PLP[26]. In the process serine is converted to glycine. Serine is also involved in the transsulfuration pathway at the steps of converting homocysteine to cystathionine and then to cysteine. Glycine is not simply a by-product of reactions involving serine, however. For example, it accepts methyl groups from SAM via GNMT, especially when SAM concentrations are high[27]. Its catabolism by the glycine cleavage system in the mitochondria generates methylene-THF with the involvement of THF, which is then used as a carbon donor in the 1C pathways[28]. The catabolism of amino acids tryptophan and histidine is of also of particular interest within 1-carbon metabolism as this produces formate[22,29]. Formate is produced in the mitochondria and then released into the cytosol, forming formyl-THF by condensation with THF. Formyl-THF can then either be used in purine synthesis or be interconverted into other THF oxidation states as part of folate metabolism. Taken together, formate, serine and glycine help link the mitochondrial and cytosolic folate pools[30].

Methionine: Methionine is obtained from the diet or formed by the methylation of homocysteine. Its adenylation forms SAM, making it a key metabolite in the 1-carbon pathways. Methionine also upregulates glycine N-methyltransferase (GNMT) which can use excess SAM to methylate glycine to sarcosine.

Homocysteine: Homocysteine is an intermediary metabolite that is hydrolysed from SAH. Since a build-up of SAH inhibits transmethylation reactions[2] homocysteine is often inversely associated with methylation. For example, van Mil *et al.* (2014) found an increase in maternal plasma homocysteine was associated with 0.04% lower methylation at *NR3C1* in the infant epigenome (p=0.03)[31]. Although it is not a nutritional exposure *per se*, nutritional inputs can influence the concentration of homocysteine. Trials that have aimed to decrease homocysteine as part of lowering blood pressure could therefore provide additional information as to exposures with the potential to influence the epigenome. For example, several studies indicate that increased consumption of ω-3 PUFAs is associated with reduced homocysteine levels[32], and that when combined with folic acid and other B vitamins the association is even stronger[33]. Betaine supplementation has also been shown to reduce homocysteine levels[27]

References

1. Lu SC, Mato JM. S-adenosylmethionine in liver health, injury, and cancer. *Physiol Rev*. 2012 Oct;**92**(4):1515–42.

2. Scotti M, Stella L, Shearer EJ, Stover PJ. Modeling cellular compartmentation in one-carbon metabolism. *Wiley Interdiscip Rev Syst Biol Med*. 2013 Jan;**5**(3):343–365.

3. Mason JB. Biomarkers of Nutrient Exposure and Status in One-Carbon (Methyl) Metabolism. *J Nutr*. 2003 Mar 1;**133**(3):941S–947.

4. Pajares MA, Pérez-Sala D. Betaine homocysteine S-methyltransferase: just a regulator of homocysteine metabolism? *Cell Mol Life Sci*. 2006 Dec;**63**(23):2792–803.

5. Fredriksen A, Meyer K, Ueland PM, Vollset SE, Grotmol T, Schneede J. Large-scale population-based metabolic phenotyping of thirteen genetic polymorphisms related to one-carbon metabolism. *Hum Mutat*. 2007 Sep;**28**(9):856–65.

6. Scott JM, Weir DG. Folic acid, homocysteine and one-carbon metabolism: a review of the essential biochemistry. *J Cardiovasc Risk*. 1998 Aug;**5**(4):223–7.

7. Selhub J. Homocysteine metabolism. *Annu Rev Nutr*. 1999 Jan 28;**19**:217–46.

8. Finkelstein JL, Layden AJ, Stover PJ. Vitamin B-12 and Perinatal Health. *Adv Nutr*. 2015 Oct 1;**6**(5):552–63.

9. Cho E, Zeisel SH, Jacques P, et al. Dietary choline and betaine assessed by food-frequency questionnaire in relation to plasma total homocysteine concentration in the Framingham Offspring Study. *Am J Clin Nutr*. 2006 Apr;**83**(4):905–11.

10. Ueland PM. Choline and betaine in health and disease. *J Inherit Metab Dis*. 2011 Feb;**34**(1):3–15.

11. Huang T, Hu X, Khan N, Yang J, Li D. Effect of Polyunsaturated Fatty Acids on Homocysteine Metabolism through Regulating the Gene Expressions Involved in Methionine Metabolism. *Sci World J*. 2013 Jan;**2013**:1–8.

12. Khot V, Kale A, Joshi A, Chavan-Gautam P, Joshi S. Expression of genes encoding enzymes involved in the one carbon cycle in rat placenta is determined by maternal micronutrients (folic acid, vitamin B12) and omega-3 fatty acids. *Biomed Res Int*. 2014 Jan;**2014**:613078.

13. DeLong CJ, Shen YJ, Thomas MJ, Cui Z. Molecular distinction of phosphatidylcholine synthesis between the CDP-choline pathway and phosphatidylethanolamine methylation pathway. *J Biol Chem*. 1999 Oct 15;**274**(42):29683–8.

14. Wijk N van, Watkins CJ, Hageman RJJ, et al. Combined dietary folate, vitamin B-12, and vitamin B-6 intake influences plasma docosahexaenoic acid concentration in rats. *Nutr Metab (Lond)*. 2012 May 30;**9**(1):49.

15. Watkins SM, Zhu X, Zeisel SH. Phosphatidylethanolamine-N-methyltransferase activity and dietary choline regulate liver-plasma lipid flux and essential fatty acid metabolism in mice. *J Nutr*. 2003 Nov;**133**(11):3386–91.

16. Selley ML. A metabolic link between S-adenosylhomocysteine and polyunsaturated fatty acid metabolism in Alzheimer’s disease. *Neurobiol Aging*. 2007 Dec;**28**(12):1834–1839.

17. Kulkarni A, Dangat K, Kale A, Sable P, Chavan-Gautam P, Joshi S. Effects of altered maternal folic acid, vitamin B12 and docosahexaenoic acid on placental global DNA methylation patterns in Wistar rats. *PLoS One*. 2011 Jan;**6**(3):e17706.

18. Roy S, Kale A, Dangat K, Sable P, Kulkarni A, Joshi S. Maternal micronutrients (folic acid and vitamin B(12)) and omega 3 fatty acids: implications for neurodevelopmental risk in the rat offspring. *Brain Dev*. 2012 Jan;**34**(1):64–71.

19. Stead LM, Brosnan JT, Brosnan ME, Vance DE, Jacobs RL. Is it time to reevaluate methyl balance in humans? *Am J Clin Nutr*. 2006 Jan;**83**(1):5–10.

20. Bertolo RF, McBreairty LE. The nutritional burden of methylation reactions. *Curr Opin Clin Nutr Metab Care*. 2013 Jan;**16**(1):102–8.

21. Khot V, Chavan-Gautam P, Joshi S. Proposing interactions between maternal phospholipids and the one carbon cycle: A novel mechanism influencing the risk for cardiovascular diseases in the offspring in later life. *Life Sci*. 2015 May 15;**129**:16–21.

22. Brosnan ME, MacMillan L, Stevens JR, Brosnan JT. Division of labour: how does folate metabolism partition between one-carbon metabolism and amino acid oxidation? *Biochem J*.

2015 Dec 1;**472**(2):135–46.

23. Rios-Avila L, Coats B, Chi Y-Y, et al. Metabolite profile analysis reveals association of vitamin B-6 with metabolites related to one-carbon metabolism and tryptophan catabolism but not with biomarkers of inflammation in oral contraceptive users. *J Nutr*. 2015 Jan 1;**145**(1):87–95.

24. Rios-Avila L, Coats B, Ralat M, et al. Pyridoxine supplementation does not alter in vivo kinetics of one-carbon metabolism but modifies patterns of one-carbon and tryptophan metabolites in vitamin B-6-insufficient oral contraceptive users. *Am J Clin Nutr*. 2015 Sep 1;**102**(3):616–25.

25. Silva VR da, Rios-Avila L, Lamers Y, et al. Metabolite profile analysis reveals functional effects of 28-day vitamin B-6 restriction on one-carbon metabolism and tryptophan catabolic pathways in healthy men and women. *J Nutr*. 2013 Nov 1;**143**(11):1719–27.

26. Davis SR, Stacpoole PW, Williamson J, et al. Tracer-derived total and folate-dependent homocysteine remethylation and synthesis rates in humans indicate that serine is the main one-carbon donor. *Am J Physiol Endocrinol Metab*. 2004 Feb;**286**(2):E272-9.

27. Olthof MR, Verhoef P. Effects of betaine intake on plasma homocysteine concentrations and consequences for health. *Curr Drug Metab*. 2005 Feb;**6**(1):15–22.

28. Nijhout HF, Reed MC, Lam S-L, Shane B, Gregory JF, Ulrich CM. In silico experimentation with a model of hepatic mitochondrial folate metabolism. *Theor Biol Med Model*. 2006;**3**:40.

29. Morrow GP, MacMillan L, Lamarre SG, et al. In vivo kinetics of formate metabolism in folate-deficient and folate-replete rats. *J Biol Chem*. 2015 Jan 23;**290**(4):2244–50.

30. Lamarre SG, Morrow G, Macmillan L, Brosnan ME, Brosnan JT. Formate: an essential metabolite, a biomarker, or more? *Clin Chem Lab Med*. 2013 Mar 1;**51**(3):571–8.

31. Mil NH van, Bouwland-Both MI, Stolk L, et al. Determinants of maternal pregnancy one-carbon metabolism and newborn human DNA methylation profiles. *Reproduction*. 2014 Dec 12;**148**(6):581–92.

32. Huang T, Zheng J, Chen Y, Yang B, Wahlqvist ML, Li D. High consumption of Ω-3 polyunsaturated fatty acids decrease plasma homocysteine: a meta-analysis of randomized, placebo-controlled trials. *Nutrition*. 2011 Sep;**27**(9):863–7.

33. Dawson SL, Bowe SJ, Crowe TC. A combination of omega-3 fatty acids, folic acid and B-group vitamins is superior at lowering homocysteine than omega-3 alone: A meta-analysis. *Nutr Res*. 2016 Jun;**36**(6):499–508.
