# Supplementary material for: Candidate genes linking maternal nutrient exposure to offspring health via DNA methylation: a review of existing evidence in humans with specific focus on one-carbon metabolism
Source: Int J Epidemiol. 2018 Aug 17;47(6):1910–37. doi: 10.1093/ije/dyy153 (PMC6280938; doi:10.1093/ije/dyy153)
Supplement: Supplementary Material 2 [file dyy153_supplementary_material_2.pdf]

Supplementary Material 2: Search terms used for narrative review

Inclusion criteria for the review:

1) Type of exposure: Nutrition-related, prioritising nutrients related to one-carbon metabolism
2) Timing of study: Preconception and pregnancy
3) Type of study: Human
4) Type of study: Intergenerational
5) Mechanism: DNA methylation
6) Outcome: growth-related, cardio-metabolic, cognitive

Databases searched: PubMed, Google Scholar. Search terms were applied to titles and abstracts.

Example of PubMed search terms:

1) **Nutritional Exposure:** (nutrition* OR micronutrient OR vitamin OR multivitamin OR folate OR "folic acid" OR B12 OR B6 OR B2 OR riboflavin OR "pyridoxal 5-phosphate" OR cobalamin OR betaine OR choline OR PUFA* OR "polyunsaturated fatty acids" OR "UNIMMAP" OR "one carbon" OR "1-carbon" OR "methyl donor" OR famine OR carbohydrate OR protein OR fat OR "energy restriction" OR "energy intake" OR diet OR supplement*)

2) **Exposure timing:** (pregnan* OR periconception* OR peri-conception* OR trimester OR "in utero")

3) **Human study:** (women OR woman OR maternal OR human) NOT (mouse OR mice OR rodent* OR rat OR rats OR pig OR pigs OR sheep)

4) **Intergenerational study:** (offspring OR infant OR child OR children OR intergeneration*)

5) **Mechanism of DNA methylation:** (epigenetic* OR epigenom* OR "DNA methylation" OR methylation)

6) **Outcome:** (outcome OR phenotype OR cardio-metabolic OR "lipid profile" OR cholesterol OR "metabolic syndrome" OR "type-2 diabetes" OR "type-II diabetes" OR "body composition" OR "bone composition" OR adipos* OR obesity OR obese OR "body mass index" OR BMI OR growth OR anthropometr* OR "blood pressure" OR "mental health" OR cognit* OR ADHD OR "attention-deficit/hyperactivity disorder" OR "intelligence quotient")

Search Strategy:

- Search 1: For the review of nutritional exposures in pregnancy on infant DNA methylation search terms from 1) to 5) were linked together with 'AND'.
- Search 2: For the review of offspring methylation patterns and phenotypic outcomes there were two approaches. Firstly, the search terms from 4) to 6) were linked together with 'AND'. Secondly, a nutrition-sensitive locus found in the first review (e.g. '*LEP*') was entered and linked with search terms 5) and 6) using 'AND'. Only genes that had featured in the first search were included in the second search.
- Search 3: To check for studies linking maternal nutritional exposure to offspring phenotype, mediated by DNA methylation, we ran the search terms from 1) to 6) linked by 'AND'.
