# Supplementary material for: Candidate genes linking maternal nutrient exposure to offspring health via DNA methylation: a review of existing evidence in humans with specific focus on one-carbon metabolism
Source: Int J Epidemiol. 2018 Aug 17;47(6):1910–37. doi: 10.1093/ije/dyy153 (PMC6280938; doi:10.1093/ije/dyy153)
Supplement: Supplementary Table 1 [file dyy153_supplementary_table_1.pdf]

Supplementary Table 1: Studies investigating associations between maternal nutritional exposures and offspring DNA methylation (Search 1)

KEY: Intervention studies | Studies using broader nutritional exposures | No colour: observational studies involving one-carbon metabolites

Reference	Exposure	Exposure timing	Study design	Genes Investigated	Summary of results
Amarasekera M, et al. *FASEB J.* 2014;**28:** 4068–76[1]	Maternal serum folate. High and low folate groups.	3rd trimester	Nested cohort study. 23 mother-infant pairs, Australia.<br>Tissue: Cord blood CD4+ antigen presenting cells.<br>Platform: Sequenom Epityper	*ZFP57* (main focus)<br><br>Other DMRS: *ACADM, C21orf56, FZD7, LASP1, LY6E,WNT9A*	High folate group: Hypomethylation at *ZFP57* (mean differential methylation 19%), *LY6E* (8%) and *C21orf56* (11%).<br>Hypermethylation at *LASP1* (8%), *ACADM* (13%), *WNT9A* (10%), *FZD7* (8%)
Amarasekera M, et al. *Epigenetics*. 2014;**9**:1570–1576[2]	Maternal PUFA supplementation	2nd & 3rd trimesters	Subset of randomised controlled trial study, (70 mother-infant pairs, 36 intervention, 34 control). Trial was 3.7 g of fish oil (56.0% as DHA and 27.7% as EPA) from 20 weeks gestation to delivery vs. control.<br>Tissue: Isolated CD4+ cells from cord blood<br>Platform: Illumina Infinium HumanMethylation450 BeadChip	*Genome-wide*	Maternal docosahexaenoic acid (DHA), eicosapentaenoic acid (EPA), or total n-3 PUFAs showed dose-response effects on methylation at certain loci but none reached genome-wide significance.
Azzi S, et al. *Epigenetics*. 2014;**9:** 338–45[3]	Maternal vitamins pre-pregnancy BMI, vitamins B2, B3, B6, B9 (folate), B12	3 months before conception and last trimester	Nested cohort study in France (254 mother-infant pairs). Healthy infants from the 'EDEN' cohort. Maternal FFQs at 15 weeks gestation and post-delivery. Pre-pregnancy weight was by recall.<br>Tissue: Infant cord blood measured<br>Platform: Allele-specific methylated multiplex real-time quantitative PCR.	*PLAGL1 (ZAC1)*	Maternal B2 was positively correlated with *ZAC1* DMR methylation index at pre-pregnancy ($r = 0.14$, $p = 0.04$) and in the last trimester ($r = 0.11$, $p = 0.09$). Pre-pregnancy BMI was positively associated with methylation at *ZAC1* DMR.
Ba Y, et al. *Eur J Clin Nutr.* 2011;**65:** 480–5[4]	Maternal serum folate and vitamin B12	Delivery	Hospital-based cross-sectional study. 99 mother-infant pairs, China.<br>Tissue: Cord blood<br>Platform: Power SYBR Green PCR Master Mix and real-time methylation specific PCR.	*IGF2*	Higher B12 associated with lower methylation at *IGF2*P3 (0.2% per SD). No association of folate with methylation.
Cooper WN, et al. *FASEB J.* 2012;**26:** 1782–90[5]	UNIMMAP supplementation	Periconception	Sub-sample from an RCT, The Gambia (58 mother-infant pairs; 36 intervention, 22 control). Intervention was one tablet of UNIMMAP vs. control from pre-pregnancy to mean 9.5 weeks gestation. UNIMMAP includes	*GNASAS, IGF2, IGF2R, MEG3 (GTL2), MEST (PEG1), PEG3, PLAGL1 (ZAC1)*	There were no overall effects of UNIMAPP supplementation. Cord blood results: In girls of supplemented mothers there was 8.6% lower methylation of *IGF2R* DMR compared to controls ($p=0.023$). In boys of supplemented mothers there

Reference	Exposure	Exposure timing	Study design	Genes Investigated	Summary of results
			15 micronutrients (vitamins A, D, E, B1, B2, B6, Bi 2, C, Niacin, Folic Acid, Fe, Zn, Cu, I, Se) at the Recommended Daily Allowance level, except for folic acid, which is included at a level of 400 µg<br><br>Tissue: Infant cord blood and venous blood at 9 months<br>Platform: mass spectrometry (SpectroCHIPs, Sequenom)		was 6.5% lower methylation of *GTL2* DMR2 compared to controls (p=0.044).<br><br>9 month venous blood results: In girls of supplemented mothers there was 7.7% lower methylation of *PEG1* compared to controls (p=0.030), and 5.5% lower methylation of *GNASAS* (p=0.047).
Dominguez-Salas P, et al. *Nat Commun*. 2014;**5:** 3746[6]	1-carbon metabolites / season	Periconception	Cohort study, The Gambia. Followed 84 women conceiving in peak of rainy season and 83 in peak of dry season and their infants.<br>Maternal measurement: plasma folate, B6, B12, active B12, choline, betaine, methionine, homocysteine, SAM, SAH, dimethyl glycine and erythrocyte B2 back-extrapolated to conception.<br>Tissue: Infant venous blood taken at mean 3.6 months of age.<br>Platform: PSQTM HS 96 pyrosequencer (Biotage)	*RBM46, BOLA3, FLJ20433 (EXD3), LOC654433 (PAX8-AS1), ZFYVE28, ZNF678*	Methylation potential in maternal plasma was higher during the rainy season compared to the dry season. Offspring of rainy season conceptions had higher levels of CpG methylation at the six metastable epialleles in peripheral blood monocytes when considered jointly. Individually only *RBM46* demonstrated a significant difference.
Drake AJ, et al., *Clin Endocrinol*. 2012;**77:**808-15[7].	Maternal diet (food groups)	'Early' (<20 weeks) and 'late' (>20 weeks) pregnancy	Retrospective cohort study ('Motherwell'). 34 offspring at 40 years of age and mother's dietary records looked up.<br>Tissue: Whole blood<br>Platform: Pyrosequencing using PSQTM HS-96A (Qiagen)	*HSD2, NR3C1, IGF2, H19 ICR, GR* exon 1F	Higher methylation at *GR* exon 1F was observed in mothers having higher meat/fish/vegetables and lower bread/potato intake in late pregnancy. Higher methylation at *HSD2* with increased meat and fish intake.
Finer S, et al. *BMJ Open.* 2016;**6:**e011768[8].	Famine	All trimesters	Case-control study, Bangladesh. Cases: Offspring exposed to famine *in utero* for at least 7 months of gestation (n=13). Controls*:* unexposed (n=18).<br><br>Tissue: Whole blood age 28-31 years	*VTRNA2-1, PAX8, PRDM9, HLA-DQB2, PLD6, near ZFP57, AKAP12, ATP5B, LRRC14B, SPG20, near BOLA, RBM46, ZFYVE28, EXD3,*	Comparing cases with unexposed controls in Bonferroni- corrected post-hoc pairwise comparisons there was higher methylation at *PAX8* (mean beta 0.771 vs. 0.730, p<0.005), lower methylation at *PRDM9* (0.656 vs 0.720, p<0.001) and lower methylation near *ZFP57* (0.681 vs. 0.683, p<0.001).

Reference	Exposure	Exposure timing	Study design	Genes Investigated	Summary of results
			Platform: Illumina Infinium HumanMethylation450 BeadChip	*PARD6G, ZNF678* and *ZFYVE28*	
Godfrey KM, et al. *Diabetes*. 2011;**60:** 1528–1534[9]	Maternal carbohydrate intake	2nd trimester	Cohort, UK (78 mother-child pairs from Princess Anne Hospital and 239 mother-infant pairs in replication cohort - Southampton Women's Study). Nutrition exposure measured by FFQ.<br><br>Tissue: Infant cord blood<br>Platform EpiTyper software v1.0 (Sequenom)	*RXRA, NOS3, SOD1, IL8, PI3KCD*	*RXRA* methylation was inversely associated with maternal carbohydrate intake in early pregnancy.
Gonseth S, et al. *Epigenetics* 2015;**10**:1166–76[10]	Maternal folate intake	Periconception	Used healthy controls from an existing case-control study. N=343, USA. Maternal folate at conception assessed by retrospective FFQs, median 4 years later. Top hits were validated in an independent dataset.<br>Tissue: Cord blood dried blood spots<br>Platform: Illumina Infinium HumanMethylation450 BeadChip	*STX11, OTX2, TFAP2A, CYS1*	All CpG methylation in the four top hits showed an inverse relationship with folate intake. Lower methylation at these sites were associated with increased gene expression.<br>When stratified by folate intake category, women with low intake (<200 µg/day) showed more positive associations between folate and methylation than inverse ones using epigenome-wide results. For women with high folate intakes (>600 µg/day) this trend reversed and more inverse associations were seen.
Haggarty P, et al. *Am J Clin Nutr* 2013; **97**:94–9[11]	Maternal folate and folic acid supplementation	Throughout pregnancy	Cohort study. 913 mother-infant pairs, UK Maternal folic acid supplementation assessed by FFQ covering pre-conception, <12 weeks gestation and >12 weeks gestation. Maternal folate was measured using red blood cell folate at 19 weeks gestation.<br>Tissue: Infant cord blood<br>Platform: Pyrosequencing (PyroMark, Qiagen).	*PEG3, IGF2 DMR, SNRPN*	Maternal folic acid supplementation started after 12 weeks gestation was associated with increased methylation in *IGF2* (0.7%, p=0.044) and decreased methylation in *PEG3* (-0.5%, p=0.018) compared to no supplementation. There was no effect of folic acid taken preconception or <12 weeks gestation. There was no association when the same analysis was done using maternal red blood cell folate.
Heijmans BT, et al. *Proc Natl Acad Sci USA*. 2008;**105:** 17046–9[12]	Famine	Throughout pregnancy	Dutch hunger winter. Retrospective case-control study six decades after famine (cases n=60 exposed to famine periconceptionally, n=62 exposed in late gestation, each case had a same-sex, unexposed sibling control).<br>Tissue: Adult whole blood	*IGF2*	In those offspring exposed to famine periconceptionally there was lower methylation at *IGF2* DMR (-5.2%, p= 5.9×10^{-5}) compared to controls. There was no association with those exposed during late gestation.

Reference	Exposure	Exposure timing	Study design	Genes Investigated	Summary of results
			Platform: a mass spectrometry–based method (Epi- typer, Sequenom).		
Hoyo C, et al. *Epigenetics*. 2011;**6:** 928–936[13]	Maternal folic acid intake ('none', 'moderate' and 'high' groups).	Preconception & Prenatal (all trimesters)	Cohort study ('NEST' cohort). 438 mother-infant pairs, USA.<br>Tissue: Cord blood leukocytes<br>Platform: Pyromark Q96 MD Pyrosequencer (Qiagen).	*IGF2, H19*	No association of pre-conception folic acid intake with *IGF2* DMR methylation.<br>'Moderate' intake associated with 2% lower methylation at *H19* compared with 'none' group. 'High' intake associated with 2.8% lower methylation. Associations stronger in boys. Similar results for pre-natal folate intakes.
Hoyo C, et al. *Epigenetics*. 2014;**9:** 1120–30[14]	Maternal erythrocyte folate	1st trimester (median 12 weeks gestation)	Cohort study ('NEST' cohort). 496 mother-infant pairs, USA.<br>Tissue: Cord blood leukocytes.<br>Platform: Pyromark Q96 MD Pyrosequencer (Qiagen).	*IGF2, H19, PEG1/MEST, PEG3, PLAGL1, MEG3-IG, PEG10/SGCE, NNAT, DLK1/MEG3*	Higher folate levels associated with decreased methylation at *MEG3, PLAGL1* and *PEG3* and increased methylation at *IGF2*.
Jiang X, et al. *FASEB J.* 2012;**26:** 3563–74[15]	Maternal serum choline	3rd trimester	Randomised controlled trial, USA (24 mother-infant pairs, 12 per arm). 480 vs. 930 mg choline / day given from 26-29 weeks gestation for 12 weeks.<br>Tissue: Placenta & cord blood leukocytes<br>Platform: EpiTyper (Sequenom).	*CRH, GNASAS, IGF2, IL10, LEP, NR3C1*	*CRH*: Higher maternal choline intake associated with higher methylation of placental promoter (~4%, p=0.05) but lower methylation (~2.5%, p=0.04) of cord blood (effect size estimated from figure).<br>*GNASAS*, *IGF2*, *IL10*, *LEP*: no effect<br>*NR3C1*: Higher maternal choline intake associated with higher methylation of placental promoter (0.7%, p=0.002) but lower methylation (-0.6%, p=0.04) of cord blood.
Joubert BR, et al. *Nat Commun.* 2016;**7:** 10577[16]	Maternal plasma folate	2nd trimester (median 18 and 13 weeks gestation for the two cohorts).	Meta-analysis of two cohorts: Norwegian Mother and Child Cohort Study ('MoBa', N=1275); Generation R (Netherlands, N=713).<br>Tissue: Infant cord blood<br>Platform: Illumina Infinium HumanMethylation450 BeadChip	*APC2, GRM8, KLK4, LHX1, OPCML, PRPH, PRSS21, SLC16A12*	443 CpGs demonstrated differential methylation by folate levels using false discovery rate (FDR) of 5%. 94% showed an inverse relationship between folate and methylation. 48 CpGs demonstrated differential methylation by folate levels after Bonferroni correction ($P<1.17 \times 10^{-7}$). The genes listed here represent the ones with the smallest p values.
Kühnen P, et al. *Cell Metab.* 2016;**24**:502–509[17]	1-carbon metabolites / season	Periconception	Cohort study, The Gambia. 144 mother-child pairs from 'MDEG' cohort.<br>Tissue: Infant venous blood taken at mean 3.6 months of age.	*POMC*	Lower methylation at the *POMC* VMR in children conceived in the dry season compared to those conceived in the rainy season (coefficient -0.152, p=0.034).

Reference	Exposure	Exposure timing	Study design	Genes Investigated	Summary of results
			Platform: Illumina Infinium HumanMethylation450 BeadChip used for initial screen.		
Lee H-S, et al. *Physiol Genomics*. 2014;**46:** 851–7[18]	Maternal PUFA supplementation	2nd & 3rd trimester	Randomised controlled trial. 261 mother-child pairs (131 supplemented, 130 control) in Mexico. 400mg docosahexaenoic acid (DHA) vs. placebo given daily from 18-22wk gestation to birth.<br>Tissue: Infant cord blood<br>Platform: PyroMark ID system (Qiagen)	*H19, IGF2*	No overall differences. The DHA supplemented group infants had lower *H19* methylation (-1.66%, p=0.01) among mothers of BMI < 25 kg/m^2. Methylation of *IGF2* DMR was higher (1.35%, p=0.03) in the supplemented group for infants born to overweight mothers. DNA methylation levels in *IGF2* P3 were higher (1.88%, p=0.04) in the DHA group than the control group in preterm infants.
Lin, X, et al. *BMC Med.* 2017;15:50[19].	Maternal BMI, glucose, plasma fatty acids, plasma vitamin D, serum B12, B6, folate, iron, zinc, magnesium	3rd trimester (26-28 weeks)	Cohort study ('GUSTO' cohort), 987 mother-infant pairs, Singapore.<br>Tissue: Infant cord blood<br>Platform: Illumina Infinium HumanMethylation450 BeadChip	EWAS	Methylation of cg25685359 (*MIRLET7BHG*) was inversely associated with maternal n-6 PUFA levels ($P = 4.2 \times 10^{-4}$).<br>Methylation of cg23671997 (IGDCC4) was positively associated with maternal fasting glucose levels ($P = 2.7 \times 10^{-4}$).
Marchlewicz EH, et al. *Sci Rep*. 2016;**6**:34857[20].	Maternal metabolomic profile, including PUFAs	1st trimester	Cohort study, USA (40 mother-infant pairs)<br>Tissue: Infant cord blood<br>Platform: PyroMark Q96 MD pyrosequencing (Qiagen).	*IGF2, H19, ESR1, PPARα*	Several derivatives of linoleic acid and α-linolenic acid were associated with candidate gene methylation, but direction of associations differed. Very Long Chain Fatty Acids were positively correlated with methylation at *ESR1* and *PPARα.*
McCullough LE, et al. *Clin Epigenetics*. 2016;**8:** 8[21]	Maternal plasma B12, B6, Hcy	1st trimester (mean 12 weeks gestation)	USA (496 mother-infant pairs), 'NEST' cohort<br>Tissue: Infant cord blood<br>Platform: Pyrosequencing (Pyromark Q96 MD, Qiagen).	*H19, MEG3 (GTL2), PEG10 / SGCE, PLAGL1 (ZAC1)*	No association between maternal micronutrient levels and *H19*, *PEG10 / SGCE, PLAGL1 (ZAC1)*<br>Vitamin B6 (PLP) in quartiles positively associated with methylation at *MEG3* DMR (βQuartile 3 = 2.01 and βQuartile 4 = 3.24, p <0.05).
McKay JA, et al. *PLoS One*. 2012;**7:** e33290[22]	Maternal red blood cell folate, serum vitamin B12	1st trimester (mean 10.6 weeks gestation)	Nested cohort study, UK (121 mother-infant pairs). Also looked at genetic variants.<br>Tissue: Infant cord blood<br>Platform: Pyrosequencing (Pyromark MD, Qiagen).	*IGFBP3, IGF2*	Methylation of the *IGFBP3* locus inversely correlated with infant cord vitamin B12 concentration (standardised β -0.345, p<0.001), but not maternal nutrient concentrations.

Reference	Exposure	Exposure timing	Study design	Genes Investigated	Summary of results
					Maternal B12 concentration inversely correlated with infant global DNA methylation.<br>Maternal MTHFR C677T genotype associated with *IGF2* methylation (but no association with micronutrients).
Pauwels S, et al. *Clin Epigenetics* 2017;**9**:16[23]	Maternal choline, betaine, folate, methionine, folic acid	Throughout pregnancy, including periconception	Cohort study ('MANOE' study). 114 mother-infant pairs, Belgium. Maternal intakes assessed by food frequency questionnaires (FFQs) at different stages of pregnancy (periconception, 1st, 2nd, 3rd trimester) for different mothers.<br>Tissue: Buccal epithelial cell in 6 months old infants<br>Platform: Pyrosequencing (PyroMark Q24, Qiagen).	*IGF2 DMR, DNMT1, LEP, RXRA*	Periconceptional maternal intake (n=21): At *RXRA* (all CpGs) each increase in 100 µg folate was associated with 0.685% increase in methylation (p=0.027), and a 0.875% increase in methylation per 100 mg increase in betaine. At *LEP* (all CpGs) each increase in 100 µg folate was associated with a decrease in 1.233% methylation (p=0.030). At *IGF2* DMR (all CpGs) an increase in 100 µg folic acid was associated with a decrease of 0.706% methylation (p=0.010)<br>2nd trimester intake (n=85): At *DNMT1* (all CpGs) an increase in 100 µg folic acid was associated with a decrease of 0.027% methylation (p=0.020)<br>3rd trimester intake (n= 82): At *DNMT1* CpG1 an increase in 100mg choline was associated with an increase of 0.156% methylation (p=0.017). At *DNMT1* CpG3 an increase of 100 µg folate was associated with an increase of 0.131% methylation (p=0.026).
Pauwels S, et al. *Epigenetics* 2017;**12**:1–10[24]	Maternal choline, betaine, folate, methionine, folic acid	Throughout pregnancy, including periconception	Cohort study ('MANOE' study). 115 mother-infant pairs, Belgium. Maternal intakes assessed by FFQ at different stages of pregnancy (periconception, 1st, 2nd, 3rd trimester) for different mothers.<br>Tissue: Cord blood<br>Platform: Pyrosequencing (PyroMark Q24 instrument, Qiagen).	*IGF2 DMR, DNMT1, LEP, RXRA*	Periconceptional maternal intake (n=24): At *DNMT1* CpG4 each increase in 100 mg betaine was associated with 0.675% increase in methylation (p=0.039). At *LEP* CpG each increase in 100 mg methionine was associated with 0.427% increase in methylation (p=0.048).<br>2nd trimester (n=89): At *LEP* CpG2 each increase in 100 mg betaine was associated with 0.575% decrease in methylation (p=0.05). Each increase in 100 µg folate was associated with 0.507% decrease in methylation (p=0.009). At *DNMT1* CpG4 each increase in 100 mg choline was associated with 0.301% decrease in methylation (p=0.031). Each increase in 100 µg folate was associated with 0.226% decrease in methylation (p=0.045).

Reference	Exposure	Exposure timing	Study design	Genes Investigated	Summary of results
					3rd trimester (n=83): At *DNMT1* CpG2 each increase in 100 mg choline was associated with 0.291% increase in methylation (p=0.022). At *RXRA* CpG2 each increase in 100 µg folate was associated with 1.0% decrease in methylation (p=0.041).<br><br>There was 2.3% higher methylation at *LEP* (mean of CpGs) when women had taken folic acid for 6 months prior to conception compared to those not taking any supplementation. Mean *RXRA* methylation was 1.2% higher in mothers who took folic acid supplementation throughout pregnancy compared to those who stopped in the 2nd trimester (p=0.01).
Qian YY, et al., J *Hum Nutr Diet*. 2016;**29**:643-51[25].	Folic Acid	Pregnancy – no details	Case-control study. 39 small-for-gestational age (SGA) infants and 49 appropriate-for-gestational-age (AGA) controls, China. Information on folic acid supplementation taken by mothers obtained through questionnaire responses.<br>Tissue: Umbilical cord blood<br>Platform: EpiTYPER, Sequenom.	*MEST,H19*	Six sites in the *H19* DMR were found to have significantly higher methylation in SGA when compared to AGA. The associations were stronger in males in the folic acid supplemented group.
Rijlaarsdam J, et al., *J Child Psychol Psychiatry*. 2017;**58:**19-27[26].	High-fat and -sugar diet	3rd trimester (32 weeks)	Nested cohort study (‘ALSPAC’ cohort). 164 youth with low conduct problems (CP) and early onset persistent (EOP) ADHD. FFQs administered to mothers.<br>Tissue: Cord blood, peripheral blood at age 7<br>Platform: Illumina Infinium HumanMethylation450 BeadChip	*IGF2*	High-fat and -sugar diet taken prenatally was associated with higher *IGF2* methylation.
Silver MJ, et al. *Genome Biol*. 2015;**16:** 118[27]	1-carbon metabolites / season	Periconception	Cohort study, The Gambia. 110 children conceived in peak of dry season, 105 conceived in peak of rainy season. Used an epigenome-wide screen to create a list of potential MEs susceptible to periconceptional environment.<br>Tissue: Infant venous blood taken at mean 3.6 months of age.	*VTRNA2-1*	The epigenome-wide screen top hit for differential methylation by season of conception was *VTRNA2-1*. Offspring conceived in the dry season had increased levels of hypomethylation (<40%) of *VTRNA2-1* DMR (p=0.004).

Reference	Exposure	Exposure timing	Study design	Genes Investigated	Summary of results
			Platform: 450k (Illumina) used for initial screen.		
Steegers-Theunissen RP, et al. *PLoS One*. 2009;4: e784[28]	Maternal folic acid	Periconception	Cross sectional study amongst controls for another study ('HAVEN'). 120 mother-infant pairs, The Netherlands. Folic acid use ascertained from questionnaires at 17 months post-delivery to determine two groups: 400 ug periconceptional supplementation (n=86) and unsupplemented (n=34).<br>Tissue: Infant blood taken at 17 months.<br>Platform: Mass spectrometry based platform (Epityper, Sequenom)	*IGF2*	Relative methylation of *IGF2* DMR (from 5 CpGs) 4.5% higher in children born to supplemented mothers (p=0.014).
Tobi EW, et al. Hum *Mol Genet*. 2009;**18:** 4046–53[29]	Famine	Periconception, late gestation	Dutch hunger winter. Retrospective case-control study six decades after famine (cases n=60 exposed to famine periconceptionally, n=60 same-sex, unexposed sibling controls). Also cases exposed to famine in late gestation (n=62) and controls (n=62).<br>Tissue: Adult whole blood<br>Platform: a mass spectrometry–based method (Epi- typer, Sequenom).	*ABCA1, CRH, GNASAS, IGF2R, IL10, INSIGF, LEP, MEG3 (GTL2)*	Famine exposure was significantly associated with methylation at *INSIGF* (↓), *GNASAS* (↑), *MEG3* (↑), *IL10* (↑), *LEP* (↑) and *ABCA1* (↑). Effect sizes ranged from -1.6 % to + 2.4 % (all p values <0.017). In addition, *INSIGF*, *GNASAS* and *LEP* demonstrated differences in methylation by sex. There was lower methylation at *GNASAS* for those exposed to famine in late gestation.
Tobi EW, et al. *PLoS One.* 2012;**7:**e37933[30]	Famine	Periconception	Dutch hunger winter. Retrospective case-control study six decades after famine (cases n=60 exposed to famine periconceptionally, n=60 same-sex, unexposed sibling controls)<br>Tissue: Adult whole blood<br>Platform: a mass spectrometry–based method (Epi- typer, Sequenom).	*INS, INSIGF, IGF2* and *H19*	Methylation at *INSIGF* was 1.5% lower in the cases compared to controls. Directions of associations with methylation at *IGF2* varied according to the specific DMR.
Tobi EW, et al. *Int J Epidemiol.* 2015 May 5;44:1211–1223[31]	Famine	Gestational weeks 1–10, 11–20, 21–30, or 31 to delivery	Dutch hunger winter. 384 subjects exposed in the four timing windows.<br>Tissue: Adult whole blood<br>Platform: Illumina Infinium HumanMethylation450 BeadChip	*FAM150B, SLC38A2, PPAP2C, OSBPL5/MRGPRG, TMEM105, TACC1* and *ZNF385A*	Famine exposure in gestational weeks 1-10 had increased methylation at *FAM150B, SLC38A2, PPAP2C*, and lower methylation at *OSBPL5/MRGPRG* compared to later windows of exposure. Methylation of *TACC1* and *ZNF385A* was increased after exposure during any time in gestation.

Reference	Exposure	Exposure timing	Study design	Genes Investigated	Summary of results
					Methylation at *TMEM105* was lower when exposure was at periconception.
Van Dijk SJ, et al. *Clin Epigenetics*. 2016;**8**:114[32]	Maternal PUFA supplementation	2nd & 3rd trimesters	Subset of RCT trial, Australia (n= 369 at birth; 190 intervention, 179 control). Trial was 800 mg/day DHA vs. placebo from 20 weeks gestation to delivery.<br>Tissue: Infant blood spots at delivery, and venepuncture at 5 years<br>Platform: Illumina Infinium HumanMethylation450 BeadChip	*ESYT3, SLC12A6, CCK, MAD1L1, GTF2A1L, STON1, RPS6KA2, FAM110B, PWWP2B, TRAK1, RAET1L*	The listed genes contained CpGs showing differential methylation according to intervention arm ($p<5x10^{-5}$). Directions of associations changed, but mostly DHA was inversely associated with methylation. Maximum effect size was a difference of 4.5% methylation. These trends were still seen in the samples at 5 years, although they no longer reach genome-wide significance.
van Mil NH, et al. *Reproduction*. 2014;**148:** 581–92[33]	Maternal plasma folate, plasma homocysteine (Hcy) and folic acid intake	1st trimester (mean 13 weeks)	Nested cohort study ('Generation R'). 463 mother-infant pairs, The Netherlands. Questionnaires assessed periconceptional folic acid intake.<br>Tissue: Infant cord blood<br>Platform: Mass spectrometry based platform (Epityper, Sequenom)	*H19, NR3C1, DRD4, 5-HTT, IGF2 DMR, KCNQ1OT1, and MTHFR*	No association between maternal plasma folate and folic acid intake with infant DNA methylation when exposures treated as continuous variables.<br>A 1 µmol/l increase in plasma homocysteine was associated with 0.04% lower methylation at *NR3C1* (p=0.03).<br>In folate deficient mothers (<7 nmol/l) there was lower methylation at *NR3C1* compared to folate replete mothers (≥7 nmol/l) (-0.60%, p<0.001).<br>Mothers with elevated Hcy (>11 µmol/l) showed 0.92% higher methylation at *H19* compared to those with low Hcy (≤11 µmol/l) (p=0.05).<br>There were no significant associations at any other gene regions.
Waterland RA, et al. *PLoS Genet*. 2010;**6:**e1001252[34]	Season	Periconception	Cross-sectional study taking samples from 30 children conceived in the rainy season and 30 conceived in the dry season, rural Gambia.<br>Tissue: Venous blood taken at mean 8.9 years.<br>Platform: PSQTM HS 96 pyrosequencer (Biotage)	*BOLA3, FLJ20433, PAX8 ZFYVE28, SLITRK1*	Methylation at all 5 metastable epialleles was higher in children conceived in the rainy season compared to the dry season (all p<0.03).

Abbreviations: DMR, differentially methylated region; EWAS, epigenome-wide association study; ME, metastable epiallele; RCT, randomised controlled trial; PUFA, polyunsaturated fatty acid; VMR, variably methylated region.

References

1. Amarasekera M, Martino D, Ashley S, et al. Genome-wide DNA methylation profiling identifies a folate-sensitive region of differential methylation upstream of ZFP57-imprinting regulator in humans. *FASEB J*. 2014 Sep 1;**28**(9):4068–76.
2. Amarasekera M, Noakes P, Strickland D, Saffery R, Martino DJ, Prescott SL. Epigenome-wide analysis of neonatal CD4 + T-cell DNA methylation sites potentially affected by maternal fish oil supplementation. *Epigenetics*. 2014 Dec 2;**9**(12):1570–1576.
3. Azzi S, Sas TCJ, Koudou Y, et al. Degree of methylation of ZAC1 (PLAGL1) is associated with prenatal and post-natal growth in healthy infants of the EDEN mother child cohort. *Epigenetics*. 2014 Mar 6;**9**(3):338–45.
4. Ba Y, Yu H, Liu F, et al. Relationship of folate, vitamin B12 and methylation of insulin-like growth factor-II in maternal and cord blood. *Eur J Clin Nutr*. 2011 Apr;**65**(4):480–5.
5. Cooper WN, Khulan B, Owens S, et al. DNA methylation profiling at imprinted loci after periconceptional micronutrient supplementation in humans: results of a pilot randomized controlled trial. *FASEB J*. 2012 May;**26**(5):1782–90.
6. Dominguez-Salas P, Moore SE, Baker MS, et al. Maternal nutrition at conception modulates DNA methylation of human metastable epialleles. *Nat Commun*. 2014 Jan 29;**5**:3746.
7. Drake AJ, McPherson RC, Godfrey KM, et al. An unbalanced maternal diet in pregnancy associates with offspring epigenetic changes in genes controlling glucocorticoid action and foetal growth. *Clin Endocrinol*. 2012 Dec;**77**(6):808–15.
8. Finer S, Iqbal MS, Lowe R, et al. Is famine exposure during developmental life in rural Bangladesh associated with a metabolic and epigenetic signature in young adulthood? A historical cohort study. *BMJ Open*. 2016 Nov 23;**6**(11):e011768.
9. Godfrey KM, Sheppard A, Gluckman PD, et al. Epigenetic Gene Promoter Methylation at Birth Is Associated With Child's Later Adiposity. *Diabetes*. 2011 Apr 6;**60**(5):1528–1534.
10. Gonseth S, Roy R, Houseman EA, et al. Periconceptional folate consumption is associated with neonatal DNA methylation modifications in neural crest regulatory and cancer development genes. *Epigenetics*. 2015 Dec 2;**10**(12):1166–76.
11. Haggarty P, Hoad G, Campbell DM, Horgan GW, Piyathilake C, McNeill G. Folate in pregnancy and imprinted gene and repeat element methylation in the offspring. *Am J Clin Nutr*. 2013 Jan 1;**97**(1):94–9.
12. Heijmans BT, Tobi EW, Stein AD, et al. Persistent epigenetic differences associated with prenatal exposure to famine in humans. *Proc Natl Acad Sci USA*. 2008 Nov 4;**105**(44):17046–9.

13. Hoyo C, Murtha AP, Schildkraut JM, et al. Methylation variation at IGF2 differentially methylated regions and maternal folic acid use before and during pregnancy. *Epigenetics*. 2011 Jul 27;**6**(7):928–936.

14. Hoyo C, Daltveit AK, Iversen E, et al. Erythrocyte folate concentrations, CpG methylation at genomically imprinted domains, and birth weight in a multiethnic newborn cohort. *Epigenetics*. 2014 Aug 27;**9**(8):1120–30.

15. Jiang X, Yan J, West AA, et al. Maternal choline intake alters the epigenetic state of fetal cortisol-regulating genes in humans. *FASEB J*. 2012 Aug;**26**(8):3563–74.

16. Joubert BR, Dekker HT den, Felix JF, et al. Maternal plasma folate impacts differential DNA methylation in an epigenome-wide meta-analysis of newborns. *Nat Commun*. 2016;**7**:10577.

17. Kühnen P, Handke D, Waterland RA, et al. Interindividual Variation in DNA Methylation at a Putative POMC Metastable Epiallele Is Associated with Obesity. *Cell Metab*. 2016 Sep 13;**24**(3):502–9.

18. Lee H-S, Barraza-Villarreal A, Biessy C, et al. Dietary supplementation with polyunsaturated fatty acid during pregnancy modulates DNA methylation at IGF2/H19 imprinted genes and growth of infants. *Physiol Genomics*. 2014 Dec 1;**46**(23):851–7.

19. Lin X, Lim IY, Wu Y, et al. Developmental pathways to adiposity begin before birth and are influenced by genotype, prenatal environment and epigenome. *BMC Med*. 2017 Mar 7;**15**(1):50.

20. Marchlewicz EH, Dolinoy DC, Tang L, et al. Lipid metabolism is associated with developmental epigenetic programming. *Sci Rep*. 2016 Oct 7;**6**:34857.

21. McCullough LE, Miller EE, Mendez MA, Murtha AP, Murphy SK, Hoyo C. Maternal B vitamins: effects on offspring weight and DNA methylation at genomically imprinted domains. *Clin Epigenetics*. 2016 Jan 22;**8**(1):8.

22. McKay JA, Groom A, Potter C, et al. Genetic and non-genetic influences during pregnancy on infant global and site specific DNA methylation: role for folate gene variants and vitamin B12. *PLoS One*. 2012 Jan 30;**7**(3):e33290.

23. Pauwels S, Ghosh M, Duca RC, et al. Maternal intake of methyl-group donors affects DNA methylation of metabolic genes in infants. *Clin Epigenetics*. 2017 Dec 7;**9**(1):16.

24. Pauwels S, Ghosh M, Duca RC, et al. Dietary and supplemental maternal methyl-group donor intake and cord blood DNA methylation. *Epigenetics*. 2017 Jan 2;**12**(1):1–10.

25. Qian Y-Y, Huang X-L, Liang H, et al. Effects of maternal folic acid supplementation on gene methylation and being small for gestational age. *J Hum Nutr Diet*. 2016 Oct;**29**(5):643–651.

26. Rijlaarsdam J, Cecil CAM, Walton E, et al. Prenatal unhealthy diet, insulin-like growth factor 2 gene (IGF2) methylation, and attention deficit hyperactivity disorder symptoms in youth with early-onset conduct problems. *J Child Psychol Psychiatry*. 2017 Jan;**58**(1):19–27.

27. Silver MJ, Kessler NJ, Hennig BJ, et al. Independent genomewide screens identify the tumor suppressor VTRNA2-1 as a human epiallele responsive to periconceptional environment. *Genome Biol*. 2015 Jun 11;**16**(1):118.

28. Steegers-Theunissen RP, Obermann-Borst SA, Kremer D, et al. Periconceptional maternal folic acid use of 400 microg per day is related to increased methylation of the IGF2 gene in the very young child. *PLoS One*. 2009 Jan 16;**4**(11):e7845.

29. Tobi EW, Lumey LH, Talens RP, et al. DNA methylation differences after exposure to prenatal famine are common and timing- and sex-specific. *Hum Mol Genet*. 2009 Nov 1;**18**(21):4046–53.

30. Tobi EW, Slagboom PE, Dongen J van, et al. Prenatal famine and genetic variation are independently and additively associated with DNA methylation at regulatory loci within IGF2/H19. *PLoS One*. 2012;**7**(5):e37933.

31. Tobi EW, Slieker RC, Stein AD, et al. Early gestation as the critical time-window for changes in the prenatal environment to affect the adult human blood methylome. *Int J Epidemiol*. 2015 May 5;**44**(4):1211–1223.

32. Dijk SJ van, Zhou J, Peters TJ, et al. Effect of prenatal DHA supplementation on the infant epigenome: results from a randomized controlled trial. *Clin Epigenetics*. 2016 Dec 4;**8**(1):114.

33. Mil NH van, Bouwland-Both MI, Stolk L, et al. Determinants of maternal pregnancy one-carbon metabolism and newborn human DNA methylation profiles. *Reproduction*. 2014 Dec 12;**148**(6):581–92.

34. Waterland RA, Kellermayer R, Laritsky E, et al. Season of conception in rural gambia affects DNA methylation at putative human metastable epialleles. *PLoS Genet*. 2010 Jan;**6**(12):e1001252.
