# Supplementary material for: Candidate genes linking maternal nutrient exposure to offspring health via DNA methylation: a review of existing evidence in humans with specific focus on one-carbon metabolism
Source: Int J Epidemiol. 2018 Aug 17;47(6):1910–37. doi: 10.1093/ije/dyy153 (PMC6280938; doi:10.1093/ije/dyy153)
Supplement: Supplementary Table 2 [file dyy153_supplementary_table_2.pdf]

Supplementary Table 2: Studies investigating associations between nutrition-sensitive candidate genes and phenotypes (Search 2).

Reference	Study design	Genes Implicated	Phenotype investigated	Summary of results
Azzi S, et al. *Epigenetics*. 2014;**9:** 338–45[1]	Nested cohort study in France. 254 mother-infant pairs. Healthy infants from the 'EDEN' cohort.<br>Tissue: Cord blood<br>Platform: Allele-specific methylated multiplex real-time quantitative PCR.	*PLAGL1 (ZAC1)*	Pre- and post-natal growth	*ZAC1* methylation index was positively correlated with estimated fetal weight at 32 weeks gestation (r=0.15, p=0.01). It was positively correlated with weight (r = 0.14, p=0.03) and BMI z-scores (r = 0.15, p=0.01) at age 1 year.
Bens S, et al. *Eur J Hum Genet.* 2013;**21**:838-43[2]	Case-control study. 98 SGA infants and 50 AGA controls from centres of the BMBF consortium, Germany (range 0-18 years).<br>Tissue: Whole blood<br>Platform: Pyrosequencer ID	*PLAGL1, IGF2R, GRB10, H19, IGF2, MEG3, NDN, SNRPN, NESP, NESPAS*	Size at birth	Cases showed hypomethylation at *GRB10* (n=1) and *H19* 2CTCF-binding site (n=1), and hypermethylation at *NDN* (n=1) and *IGF2* (n=1). Note case study approach therefore not included in main narrative Table 2.
Bouwland-Both MI, et al. *PLoS One.* 2013;**8:**e81731[3]	Nested cohort study ('Generation R' cohort subset). 69 small-for-gestational age (SGA) vs. 471 controls (appropriate-for-gestational-age; AGA).<br>Tissue: Cord blood<br>Platform: EpiTYPER, Sequenom	*IGF2, H19, MTHFR*	Birthweight and anthropometry. Weight gain at 3 months	Methylation at the *MTHFR* locus did not vary significantly between SGA and AGA infants. An inverse association was found between SGA and *IGF2* DMR0 methylation (β =-1.07, p=0.015). SGA was not significantly associated with *H19* promoter DMR methylation. *IGF2* DMR0 methylation was inversely associated with birth-three months weight gain (β=-0.46, p=0.022).
Burris HH, et al., *Epigenomics*. 2013; **5:** 271–281[4]	Cohort study. 219 infants, Mexico.<br>Tissue: Cord blood.<br>Platform: Pyrosequencing	*IGF2/H19, KCNQ1OT1, GCR, NR3C1, LINE-1, Alu*	Birthweight	No significant methylation-birthweight associations were found for the studied loci.
Córdova-Palomera A, et al. *PLoS One*. 2014;**9:**e103639[5]	Nested cohort study. 34 monozygotic twin pairs of European descent, Spain. Age 22-56 years.<br>Tissue: Whole blood<br>Platform: Illumina Infinium HumanMethylation450 (450K) Bead-Chip.	*IGF2, IGF2BP1, IGF2BP2, IGF2BP3*	Birthweight, working memory	Mean methylation at 2 CpG sites in *IGF2BP1* was associated with birthweight (β=83.3 x 10^{-3}, p=0.033) and working memory (β= -4.4 x 10^{-3}, p=0.009). No variation in methylation was observed in other loci studied.
Deodati A, et al. *Horm Res Paediatr.* 2013;**79:**361-7[6]	Cross-sectional study. 85 children, Italy. Mean age 11.6 years.<br>Tissue: Whole blood<br>Platform: Methyl-Profiler DNA Methylation qPCR Assay	*IGF2*	Lipid profile	Children with intermediate methylation at *IGF2* had significantly higher levels of triglycerides (107.6 ± 41.99 vs. 76.6 ± 30.18 mg/dl, p < 0.005) and higher triglyceride:high-density lipoprotein-cholesterol ratio (2.23 ± 0.98 vs. 1.79 ± 0.98, p < 0.02) in comparison with children showing hypomethylation at *IGF2*.
Drake AJ, et al. *Clin Endocrinol*. 2012;**77:**808-15[7]	Retrospective cohort study ('Motherwell'). 34 offspring at 40 years of age.	*IGF2, H19 ICR, HSD2, NR3C1*	Birthweight, current height, weight, waist	There was an inverse association between methylation at specific CpGs in *HSD2* and neonatal ponderal index (Region

Reference	Study design	Genes Implicated	Phenotype investigated	Summary of results
	Tissue: Whole blood<br>Platform: Pyrosequencing using PSQTM HS-96A (Qiagen)		circumference, blood pressure	2- CpG14 r=-0.38, CpG19 r=-0.34, p<0.05) and *H19* ICR methylation and birth length (r=-0.36, p<0.05). *HSD2* methylation was positively associated with birthweight (r=0.49, p<0.05), adiposity measures and blood pressure in adulthood. *H19* ICR methylation was not significantly associated with birthweight but was positively associated with weight in adulthood (β=0.37,p=0.03). Both *H19* and *NR3C1* Exon methylation were associated with waist circumference, BMI and blood pressure at age 40.
Dunstan J, et al., *Clin Epigenetics*. 2017; **29:**29[8]	431 adolescents, USA. Age 10-15 years.<br>Tissue: Saliva<br>Platform: Pyromark Q24, Qiagen	*LEP, ICAM-1, CRH, LINE-1*	BMI, waist circumference, percent body fat	In obese boys, *LEP* methylation was inversely associated with the obesity. No significant associations were found for *ICAM-1*, *CRH* and *LINE-1*.
García-Cardona MC, et al. *Int J Obes* 2014;**38:**1457-65[9]	Cross-sectional study, n=106 divided into lean and obese adolescents, Mexico. Age 10-16 years.<br>Tissue: Whole blood<br>Platform: Methylation-specific PCR	*LEP, ADIPOQ*	BMI, insulin resistance, glucose, cholesterol and triglycerides levels	No significant variation in overall methylation of *LEP, ADIPOQ*. Lower methylation at both genes in obese subjects with insulin resistance.
Godfrey KM, et al. *Diabetes*. 2011;**60:** 1528–1534[10]	Cohort study, UK (78 mother-child pairs from Princess Anne Hospital (PAH) and 239 mother-infant pairs in replication cohort - Southampton Women's Study(SWS)). Childhood adiposity measurements were made using dual energy X-ray absorptiometry DEXA (PAH study, age 9 years; SWS, age 6 years).<br>Tissue: Cord blood<br>Platform EpiTyper software v1.0 (Sequenom)	*RXRA, eNOS*	Adiposity at age 9 years	*RXRA* methylation was associated with sex-adjusted childhood fat mass (exponentiated regression coefficient [β] 17% per SD change in methylation, p=0.009) and % fat mass (β=10%, p=0.023) at age 9 in the PAH cohort and also had similar association in the SWS cohort (Fat mass- β=6%, p=0.002; %fat mass- β=4%, p=0.002).<br>Methylation at *eNOS* was associated with fat mass (β=20%, p<0.001) and %fat mass (β=12%, p=0.002) in the PAH cohort only.
Harvey, NC et al. *J Bone Miner Res.* 2014; **29.3:** 600–607[11]	Cohort, UK (230 Southampton Women's Study and 64 Princess Anne Hospital mother-infant pairs)<br>Tissue: Cord blood<br>Platform: Sequenom MassARRAY Compact System	*RXRA*	Bone mineral content at age 4 years	Methylation at four of six *RXRA* CpG sites inversely correlated with % bone mineral content. Note that maternal free 25(OH)-vitamin D index inversely associated with methylation at one *RXRA* CpG site (β=−3.3 SD/unit, p=0.03).
Houde AA, et al. *BMC Med Genet.* 2015;**16:**29[12]	Cross-sectional study. 73 severely obese adults, Canada. Mean age 34.7 years.<br>Tissue: Blood, subcutaneous (SAT) and visceral adipose tissues (VAT).<br>Platform: Pyrosequencing	*LEP, ADIPOQ*	BMI, anthropometry, blood pressure, lipid profile	Higher *ADIPOQ* methylation levels in SAT were associated with higher BMI and waist circumference. Lower *LEP* methylation in blood was associated with higher BMI. A positive correlation was found between fasting LDL-C

Reference	Study design	Genes Implicated	Phenotype investigated	Summary of results
				levels and *LEP* in blood and SAT, and with *ADIPOQ* in *SAT* and VAT.
Hoyo C, et al. *Cancer Causes Control*. 2012;**23:**635-45[13]	Cohort study (NEST cohort), USA. 300 mother-infant pairs.<br>Tissue: Cord blood<br>Platform: Pyromark Q96 MD (Qiagen)	*IGF2, H19*	Birthweight	*IGF2* DMR0 methylation was inversely associated with *IFG2* protein concentrations in cord blood (β = -9.87, p<0.01), having stronger associations in infants of obese women (β =-20.21, p<0.0001). Higher concentrations of *IGF2* were related to higher birth weight (p<0.001). No associations found with *H19* DMR.
Hoyo C, et al. *Epigenetics*. 2014;**9:** 1120–30[14]	Cohort study ('NEST' cohort). 438 mother-infant pairs, USA.<br>Tissue: Cord blood leukocytes<br>Platform: Pyromark Q96 MD Pyrosequencer (Qiagen).	*H19*, *PEG10/SGCE*, *PLAGL1*, *MEG3*	Birth weight	Higher methylation at *H19*, *PEG10/SGCE* and *PLAGL1* DMRs, and lower *MEG3* methylation, associated with higher birth weight.
Huang RC, et al. *Clin Epigenetics*. 2012;**4:**21[15]	Cohort study ('Raine' Study). 315 children, Australia. Anthropometric parameters measured at birth and follow ups taken at 8 time points after birth.<br>Tissue: Whole blood at age 17<br>Platform: EpiTyper, Sequenom.	*IGF2/H19*	BMI, anthropometry, birthweight	No association was noted between *IGF2/H19* ICR1 and anthropometric measures at birth. A 3.4% increase in methylation at 2 specific CpGs in the *IGF2/H19* ICR was associated with 18 mm decrease in head circumference at age 17 (p= 0.006).
Kappil MA, et al. *Epigenetics*. 2015;**10:**842-9[16]	Cohort study (Rhode Island Child Health Study (RICHS), USA). 677 mother-infant pairs, subset of n=211 for methylation analysis.<br>Tissue: Placenta<br>Platform: Infinium HumanMethylation450 BeadChip.	108 imprinted genes	Size at birth	Increased methylation of *MEST* observed in SGA infants. The paper focuses on gene expression data.
Kuehnen, P. et al. *PLoS genetics*. 2012; **8:** p.e1002543[17]	Case-control study, Germany. Normal weight controls (mean age 17.9 years, N=90), obese cases (mean age 11 years, N=171).<br>Tissue: Peripheral blood<br>Platform: Direct sequencing of bisulfite-converted DNA	*POMC*	Obesity at age 11 years	Increased methylation score at *POMC* in obese children compared to controls. Overall CpG methylation score from position -4 to +6 was 40% in cases and 25% in controls (p<0.001).
Kühnen P, et al. *Cell Metab*. 2016;**24**:502–509[18]	Case-control study in German adults: 103 normal-weight and 125 obese, mean age 48.2 years.<br>Tissue: Peripheral blood<br>Platform: Pyromark Q24 (Qiagen)	*POMC*	BMI	Positive correlation of average *POMC* VMR methylation with BMI (r = 0.18, p = 0.006).
Lesseur C, et al. *Mol Cell Endocrinol.* 2013;**381:**160-7[19]	Nested cohort study, USA. Rhode Island Child Health Study 'RICHS' cohort, 81 mother-infant pairs, categorised by birth size.<br>Tissue: Cord blood, placenta<br>Platform: Pyromark MD (Qiagen)	*LEP*	Size at birth	Higher *LEP* methylation levels were observed in SGA children ($p=4.6\times10^{-3}$). Infants born to pre-pregnancy obese mothers had lower cord blood methylation levels (p=0.03). There was an interaction between placental *LEP* methylation and sex (p=0.05).

Reference	Study design	Genes Implicated	Phenotype investigated	Summary of results
Lesseur C, et al. *Psychoneuroendocrinology*. 2014;**40:**1-9[20]	Nested cohort study, USA. Rhode Island Child Health Study 'RICHS' cohort. N=444. NICU Network Neurobehavioral Scales (NNNS) used for neurobehavior testing.<br>Tissue: Placenta<br>Platform: Pyromark MD (Qiagen)	*LEP*	Neurobehavior	In male infants increased *LEP* methylation a was associated with membership in the profile of increased lethargy and hypotonicity (OR = 1.9; 95% CI: 1.07–3.4), and reduced risk of membership in the profile of decreased lethargy and hypotonicity (OR = 0.54; 95% CI: 0.3–0.94).
Lester BM, et al. *Epigenomics*. 2015;**7:**1123-36[21]	Cross-sectional study of 67 preterm infants, USA.<br>Tissue: Buccal cells<br>Platform: Pyrosequencing	*HSD2* and *NR3C1*	Neurobehaviour	Infants with a high-risk neurobehavioral profile showed increased *NR3C1* methylation and decreased *HSD2* methylation compared to infants with a low-risk neurobehavioral profile.
Lin, X, et al. *BMC Med*. 2017 Mar 7;**15:**50[22]	Cohort study ('GUSTO' cohort), 987 mother-infant pairs, Singapore.<br>Tissue: Cord blood.<br>Platform: Illumina Infinium HumanMethylation450 BeadChip	*ANK3*, *CDKN2B*, *IGDCC4*, *P4HA3*, *MIRLET7BHG*, *CACNA1G* and *ZNF423*	Birthweight, size and adiposity at 4 years	Methylation at *ANK3*, *CDKN2B*, *IGDCC4*, *P4HA3*, *MIRLET7BHG*, *CACNA1G* and *ZNF423* were associated with birth weight. *MIRLET7BHG* was the only 'nutrition-sensitive' loci considered in this review, however.
Murphy R, et al. *BMC Med Genet*. 2014;**15:**67[23]	Cohort study (Auckland Birthweight Collaborative ('ABC') study) following 153 children out of which 80 were SGA. Age 11 years<br>Tissue: whole blood<br>Platform: Methylation-specific multiplex-ligation-dependent probe amplification assay and pyrosequencing.	*IGF2, H19, KCNQ10T1*	Size at birth	*IGF2* DMR0 methylation was 2.7% lower in SGA children. Methylation did not vary at *H19* and *KCNQ10T1* ICRs.
Paquette AG, et al. *Epigenomics*. 2015;**7:**767-79[24]	Rhode Island Child Health Study 'RICHS' cohort . N=547 infants. NICU Network Neurobehavioral Scales (NNNS) used for neurobehavior testing.<br>Tissue: Placenta<br>Platform: Pyromark MD (Qiagen)	*NR3C1, HSD11B2, FKBP5, ADCYAP1R1*	Neurobehaviour	Using maximum likelihood factor analysis, 3 factors were identified as explaining the maximum variability in DNA methylation. *NR3C1* loaded strongly onto a factor that was associated with decreased quality of movement and self-regulation, increased arousal and excitability, and increased non-optimal refluxes and stress abstinence scores, also referred to as a 'poorly regulated' profile. *HSD11B2* methylation loaded onto factor associated with reducing risk of being in the poorly regulated profile.
Qian YY, et al. *J Hum Nutr Diet*. 2016;**29**:643-51[25]	Case-control study. 39 small-for-gestational age (SGA) infants and 49 appropriate-for-gestational-age (AGA) controls, China.<br>Tissue: Umbilical cord blood<br>Platform: EpiTYPER, Sequenom.	*MEST,H19*	Size at birth	Methylation in SGA children was higher (p<0.05) than controls at 3 sites in the *H19* DMR. Six sites in the *H19* DMR had higher methylation in SGA compared to AGA, but only in only males born to mothers who supplemented in pregnancy with folic acid.

Reference	Study design	Genes Implicated	Phenotype investigated	Summary of results
Rijlaarsdam J, et al. *J Child Psychol Psychiatry*. 2017;**58:**19-27[26]	Nested cohort study ('ALSPAC' cohort). 164 youth with low conduct problems (CP) and early onset persistent (EOP) ADHD.<br>Tissue: Cord blood, peripheral blood at age 7<br>Platform: Illumina Infinium HumanMethylation450 BeadChip	*IGF2*	ADHD	In the case of EOP individuals, *IGF2* methylation was positively associated with ADHD symptoms.
Souren NY, et al., *Obesity*. 2011;**19:**1519-22[27]	Monozygotic (MZ) twins study. 16 BMI discordant MZ twin pairs recruited from the East Flanders Prospective Twin Survey (EFPTS), Belgium. Mean age 30.9 years.<br>Tissue: Saliva<br>Platform: PCR amplification followed by a single-nucleotide primer extension reaction	*KvDMR1, H19, IGF2, GRB10, MEST, SNRPN, GNAS*	BMI	Methylation differences of the studied imprinted genes in salivary DNA did not account for the discordance in BMI between twins in this cohort.
St-Pierre J, et al. *Epigenetics*. 2012;**7:**1125-32[28]	Cohort study. 50 mother-infant pairs, French-Canadian origin, Canada.<br>Tissue: Placenta<br>Platform: Pyromark Q24 (Qiagen)	*IGF2, H19*	Birthweight	*IGF2* DMR2 mean methylation on the fetal placental side was positively correlated with birthweight (Spearman rank correlation=0.44, p< 0.01), height (r=0.40, p< 0.01), head (r=0.32, p< 0.05) and thorax (r=0.32,p < 0.05) circumference. 31% variance in birth weight could be accounted for by *IGF2/H19* genotype and epigenotype jointly.
Steegers-Theunissen RP, et al. *PLoS One*. 2009;4: e784[29]	Cross sectional study amongst controls for another study ('HAVEN'). 120 mother-infant pairs, The Netherlands.<br>Tissue: Infant blood taken at 17 months.<br>Platform: Epityper, Sequenom	*IGF2*	Birth weight	*IGF2* methylation was inversely associated with birthweight (-1.7% methylation per SD birthweight; p=0.034).
Tobi EW, et al. *Epigenetics*. 2011;**6:**171-6[30]	Cohort study (The Dutch Project on Preterm and Small for Gestational Age Infants ('POPS')). 38 small for gestational age (SGA) and 75 appropriate for gestational age (AGA) individuals studied.<br>Tissue: Whole blood at age 19. Methylation analysis by Platform: Epityper, Sequenom.	*IGF2, GNASAS, INSIGF, LEP*	Size at birth	Methylation levels of *IGF2* DMR0, *GNASAS*, *INSIGF* and *LEP* did not significantly vary between SGA and AGA individuals at 19 years of age.
Wijnands KP, et al. *Nutr Metab Cardiovasc Dis.* 2015;**25**:608-14[31]	Cohort study. 120 healthy children at 17 months of age, Netherlands.<br>Tissue: Whole blood<br>Platform: EpiTyper, Sequenom.	*TNF*α, *LEP*	Lipid profile	High-density lipoprotein-cholesterol levels in the children were inversely associated with *TNFa* methylation (-6.1%, p =0.058) and *LEP* (-3.4%, p=0.021)

Abbreviations: ADHD, attention-deficit/hyperactivity disorder; BMI, body mass index; CI, confidence interval; DMR, differentially methylated region; ICR, imprinting control region; LDL-c, low-density lipoprotein cholesterol; OR, odds ratio; SD, standard deviation; VMR, variably methylated region.

References

1. Azzi S, Sas TCJ, Koudou Y, et al. Degree of methylation of ZAC1 (PLAGL1) is associated with prenatal and post-natal growth in healthy infants of the EDEN mother child cohort. *Epigenetics*. 2014 Mar 6;**9**(3):338–45.
2. Bens S, Haake A, Richter J, et al. Frequency and characterization of DNA methylation defects in children born SGA. *Eur J Hum Genet*. 2013 Aug;**21**(8):838–43.
3. Bouwland-Both MI, Mil NH van, Stolk L, et al. DNA methylation of IGF2DMR and H19 is associated with fetal and infant growth: the generation R study. *PLoS One*. 2013 Jan 12;**8**(12):e81731.
4. Burris HH, Braun JM, Byun H, et al. Association between birth weight and DNA methylation of IGF2, glucocorticoid receptor and repetitive elements LINE-1 and Alu. *Epigenomics*. 2013 Jun;**5**(3):271–81.
5. Córdova-Palomera A, Alemany S, Fatjó-Vilas M, et al. Birth weight, working memory and epigenetic signatures in IGF2 and related genes: a MZ twin study. *PLoS One*. 2014;**9**(8):e103639.
6. Deodati A, Inzaghi E, Liguori A, et al. IGF2 methylation is associated with lipid profile in obese children. *Horm Res Paediatr*. 2013;**79**(6):361–7.
7. Drake AJ, McPherson RC, Godfrey KM, et al. An unbalanced maternal diet in pregnancy associates with offspring epigenetic changes in genes controlling glucocorticoid action and foetal growth. *Clin Endocrinol*. 2012 Dec;**77**(6):808–15.
8. Dunstan J, Bressler JP, Moran TH, et al. Associations of LEP, CRH, ICAM-1, and LINE-1 methylation, measured in saliva, with waist circumference, body mass index, and percent body fat in mid-childhood. *Clin Epigenetics*. 2017 Dec;**9**(1):29.
9. García-Cardona MC, Huang F, García-Vivas JM, et al. DNA methylation of leptin and adiponectin promoters in children is reduced by the combined presence of obesity and insulin resistance. *Int J Obes*. 2014 Nov;**38**(11):1457–65.
10. Godfrey KM, Sheppard A, Gluckman PD, et al. Epigenetic Gene Promoter Methylation at Birth Is Associated With Child's Later Adiposity. *Diabetes*. 2011 Apr 6;**60**(5):1528–1534.
11. Harvey NC, Sheppard A, Godfrey KM, et al. Childhood bone mineral content is associated with methylation status of the RXRA promoter at birth. *J Bone Miner Res*. 2014 Mar;**29**(3):600–7.
12. Houde A-A, Légaré C, Biron S, et al. Leptin and adiponectin DNA methylation levels in adipose tissues and blood cells are associated with BMI, waist

girth and LDL-cholesterol levels in severely obese men and women. *BMC Med Genet*. 2015 May;**16**:29.

13. Hoyo C, Fortner K, Murtha AP, et al. Association of cord blood methylation fractions at imprinted insulin-like growth factor 2 (IGF2), plasma IGF2, and birth weight. *Cancer Causes Control*. 2012;**23**(4):635–645.

14. Hoyo C, Daltveit AK, Iversen E, et al. Erythrocyte folate concentrations, CpG methylation at genomically imprinted domains, and birth weight in a multiethnic newborn cohort. *Epigenetics*. 2014 Aug 27;**9**(8):1120–30.

15. Huang R-C, Galati JC, Burrows S, et al. DNA methylation of the IGF2/H19 imprinting control region and adiposity distribution in young adults. *Clin Epigenetics*. 2012 Nov;**4**(1):21.

16. Kappil MA, Green BB, Armstrong DA, et al. Placental expression profile of imprinted genes impacts birth weight. *Epigenetics*. 2015;**10**(9):842–9.

17. Kuehnen P, Mischke M, Wiegand S, et al. An Alu element-associated hypermethylation variant of the POMC gene is associated with childhood obesity. Yeo GSH, editor. *PLoS Genet*. 2012 Mar 15;**8**(3):e1002543.

18. Kühnen P, Handke D, Waterland RA, et al. Interindividual Variation in DNA Methylation at a Putative POMC Metastable Epiallele Is Associated with Obesity. *Cell Metab*. 2016 Sep 13;**24**(3):502–9.

19. Lesseur C, Armstrong DA, Paquette AG, Koestler DC, Padbury JF, Marsit CJ. Tissue-specific Leptin promoter DNA methylation is associated with maternal and infant perinatal factors. *Mol Cell Endocrinol*. 2013 Dec 5;**381**(1–2):160–7.

20. Lesseur C, Armstrong DA, Murphy MA, et al. Sex-specific associations between placental leptin promoter DNA methylation and infant neurobehavior. *Psychoneuroendocrinology*. 2014 Feb;**40**(1):1–9.

21. Lester BM, Marsit CJ, Giarraputo J, Hawes K, LaGasse LL, Padbury JF. Neurobehavior related to epigenetic differences in preterm infants. *Epigenomics*. 2015 Oct;**7**(7):1123–36.

22. Lin X, Lim IY, Wu Y, et al. Developmental pathways to adiposity begin before birth and are influenced by genotype, prenatal environment and epigenome. *BMC Med*. 2017 Mar 7;**15**(1):50.

23. Murphy R, Thompson JM, Tost J, Mitchell E a, Auckland Birthweight Collaborative Study Group. No evidence for copy number and methylation variation in H19 and KCNQ10T1 imprinting control regions in children born small for gestational age. *BMC Med Genet*. 2014;**15**(1):67.

24. Paquette AG, Lester BM, Lesseur C, et al. Placental epigenetic patterning of glucocorticoid response genes is associated with infant neurodevelopment. *Epigenomics*. 2015 Aug;**7**(5):767–779.

25. Qian Y-Y, Huang X-L, Liang H, et al. Effects of maternal folic acid supplementation on gene methylation and being small for gestational age. *J Hum*

Nutr Diet. 2016 Oct;**29**(5):643–651.

26. Rijlaarsdam J, Cecil CAM, Walton E, et al. Prenatal unhealthy diet, insulin-like growth factor 2 gene (IGF2) methylation, and attention deficit hyperactivity disorder symptoms in youth with early-onset conduct problems. *J Child Psychol Psychiatry*. 2017 Jan;**58**(1):19–27.

27. Souren NYP, Tierling S, Fryns J-P, Derom C, Walter J, Zeegers MP. DNA methylation variability at growth-related imprints does not contribute to overweight in monozygotic twins discordant for BMI. *Obesity*. 2011 Jul;**19**(7):1519–22.

28. St-Pierre J, Hivert M-F, Perron P, et al. IGF2 DNA methylation is a modulator of newborn's fetal growth and development. *Epigenetics*. 2012 Oct;**7**(10):1125–32.

29. Steegers-Theunissen RP, Obermann-Borst SA, Kremer D, et al. Periconceptional maternal folic acid use of 400 microg per day is related to increased methylation of the IGF2 gene in the very young child. *PLoS One*. 2009 Jan 16;**4**(11):e7845.

30. Tobi EW, Heijmans BT, Kremer D, et al. DNA methylation of IGF2, GNASAS, INSIGF and LEP and being born small for gestational age. *Epigenetics*. 2011 Feb;**6**(2):171–6.

31. Wijnands KPJ, Obermann-Borst SA, Steegers-Theunissen RPM. Early life lipid profile and metabolic programming in very young children. *Nutr Metab Cardiovasc Dis*. 2015 Jun;**25**(6):608–14.
